# Heterotypic interactions drive antibody synergy against a malaria vaccine candidate

**DOI:** 10.1038/s41467-022-28601-4

**Published:** 2022-02-17

**Authors:** Robert J. Ragotte, David Pulido, Amelia M. Lias, Doris Quinkert, Daniel G. W. Alanine, Abhishek Jamwal, Hannah Davies, Adéla Nacer, Edward D. Lowe, Geoffrey W. Grime, Joseph J. Illingworth, Robert F. Donat, Elspeth F. Garman, Paul W. Bowyer, Matthew K. Higgins, Simon J. Draper

**Affiliations:** 1 Department of Biochemistry, University of Oxford, Oxford, UK; 2 Jenner Institute, University of Oxford, Oxford, UK; 3 Bacteriology Division, MHRA-NIBSC, South Mimms, Potters Bar, Hertfordshire, UK; 4 Surrey Ion Beam Centre, University of Surrey, Guildford, UK; 5 Kavli Institute of NanoTechnology Discovery, University of Oxford, Oxford, UK

**Keywords:** Vaccines, Parasitology, Antibodies, Malaria

## Abstract

Understanding mechanisms of antibody synergy is important for vaccine design and antibody cocktail development. Examples of synergy between antibodies are well-documented, but the mechanisms underlying these relationships often remain poorly understood. The leading blood-stage malaria vaccine candidate, CyRPA, is essential for invasion of *Plasmodium falciparum* into human erythrocytes. Here we present a panel of anti-CyRPA monoclonal antibodies that strongly inhibit parasite growth in in vitro assays. Structural studies show that growth-inhibitory antibodies bind epitopes on a single face of CyRPA. We also show that pairs of non-competing inhibitory antibodies have strongly synergistic growth-inhibitory activity. These antibodies bind to neighbouring epitopes on CyRPA and form lateral, heterotypic interactions which slow antibody dissociation. We predict that such heterotypic interactions will be a feature of many immune responses. Immunogens which elicit such synergistic antibody mixtures could increase the potency of vaccine-elicited responses to provide robust and long-lived immunity against challenging disease targets.

## Abstract (web summary)

Antibodies can have synergistic effects, but mechanisms are not well understood. Here, Ragotte *et al*. identify three antibodies that bind neighbouring epitopes on CyRPA, a malaria vaccine candidate, and show that lateral interactions between the antibodies slow dissociation and inhibit parasite growth synergistically.

## Introduction

Antibody or drug synergy occurs when two agents have a combined effect that exceeds their predicted additive behaviour, defined a priori based on the effect of each as a monotherapy. The goal of combining synergistic antibodies is to enhance overall activity or to achieve equal activity at lower doses. This can occur either by combining monoclonal antibodies into cocktails to be provided through passive transfer, or by the design of vaccine immunogens that elicit synergistic polyclonal antibody mixtures. In the context of infectious disease, leveraging antibody synergy in vaccine and cocktail design could result in enhanced immunity against so-far intractable diseases of global concern, such as malaria.

Synergy has been documented between antibodies against diverse pathogens, including HIV^1^, SARS-CoV^2^, SARS-CoV-2^3^, Hepatitis C virus^4^, malaria^5–7^, and Ebola^8^. While examples of synergy between monoclonal antibody pairs are relatively widespread, there are fewer descriptions of the molecular basis for these effects. In one example, synergy between two Ebola monoclonal antibodies resulted from an antibody-induced conformational change in the surface glycoprotein, which created a binding site for a second antibody^9^. In a second example, a non-neutralizing malaria monoclonal antibody slowed erythrocyte invasion, thereby giving more time for neutralizing antibodies to bind^5^. For antibodies against antigens with repeated structures, synergy can also occur through homotypic interactions between copies of the same antibody that bind to neighbouring repeated epitopes. Such interactions can drive enhanced B cell activation, as seen in the case of antibodies targeting the sporozoite-stage malaria vaccine antigen, PfCSP^10–12^. Homotypic antibody–antibody interactions are also possible when the antigen is multimeric, as seen in the binding of therapeutic monoclonal antibody rituximab to dimeric CD20^13^. These studies show that antibody synergy can arise from a range of diverse molecular and cellular processes.

The requirement for a high-quality antibody response to prevent disease is seen clearly in the development of a vaccine against the blood stage of *Plasmodium falciparum*, the pathogen responsible for the deadliest form of malaria. Here, vaccine development has been slowed by the need for high concentrations of neutralizing antibodies to achieve protection^14^. Two possible strategies can address this: to increase the quantity of polyclonal antibody induced, or to increase its “functional quality” through the induction of a more neutralizing polyclonal antibody response. To reduce the total amount of neutralizing polyclonal antibody required to protect, individual antibody clones within the polyclonal pool must somehow be optimized for their ability to neutralize parasites. To achieve this goal, it is important to understand how different antibodies within a vaccine-induced polyclonal response cooperate or antagonize one another.

The leading vaccine candidates for a *P. falciparum* blood-stage malaria vaccine are members of a heterotrimeric complex containing reticulocyte-binding protein homolog 5 (RH5), cysteine-rich protective antigen (CyRPA), and RH5-interacting protein (RIPR), collectively called the “RCR complex”^15^. Each member of this complex is highly conserved, essential for erythrocyte invasion, and capable of inducing cross-strain neutralizing antibodies^16–18^.

Of the RCR components, vaccine development is most advanced for RH5. This is the first member of the complex to enter clinical vaccine trials. RH5 has been shown to protect non-human primates from developing malaria in a heterologous challenge model^19^ and, in a human challenge study, successfully reduced the parasite multiplication rate and delayed time to the onset of symptoms^20^. These findings highlight that RCR complex components remain compelling vaccine candidates. However, while vaccine-induced immunity against blood-stage malaria appears possible, it requires induction of a more potent antibody response, in terms of quantity or quality, than that achieved to date.

Here, we describe a panel of monoclonal antibodies (mAbs) that target CyRPA and that achieve greater parasite neutralization than other anti-CyRPA mAbs reported to date. These mAbs all bind to the same face of CyRPA and potently synergize with each other when used in combination. Finally, we demonstrate that this synergy is mediated through lateral heterotypic interactions between mAbs with adjacent epitopes, which increases their affinity for CyRPA. We propose that this mechanism is likely to be observed for antibodies against many different pathogens and should be considered when designing vaccine immunogens.

## Results

### Identifying growth-inhibitory monoclonal antibodies against CyRPA

**Fig. 1:**
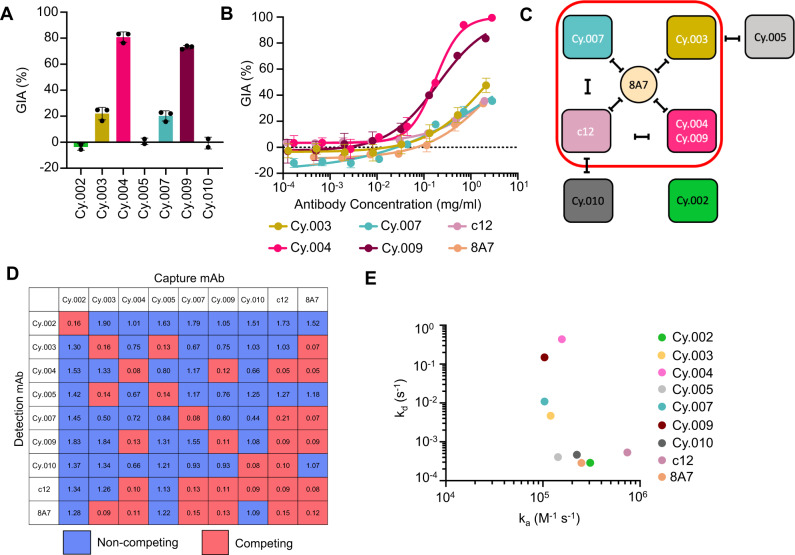
Characterization of growth inhibitory activity and binding properties of a panel of anti-CyRPA mAbs. **A** In vitro growth inhibitory activity (GIA) of monoclonal antibodies against the 3D7 clone of *P. falciparum* at 0.5 mg/ml, individual points indicate the value of each independent replicate, the bar indicates the mean, and errors bars indicate standard deviation across independent replicates (*n* = 2, Cy.002, Cy.005 and Cy.010, *n* = 3 Cy.003, Cy.004, Cy.007, Cy.009). **B** GIA dilution curve starting at 2 mg/ml of each inhibitory antibody, including chimeric human c12 and 8A7. Points show mean of triplicates and error bars indicate standard deviation. Curve fit used a four-parameter dose-response curve with the upper bound constrained to 100% GIA. All GIA curves were repeated at least twice with a single representative dilution curve shown here. **C** Competing interactions between mAbs based on the data in panel (**D**). Red box contains inhibitory mAbs and black lines indicate mAbs that compete with one another. **D** Competition matrix of all nine anti-CyRPA mAbs. The value contained in each box is the mean OD_405_ of a given mAb pair across triplicate; a value <0.25 was taken to be negative binding of the detection mAb. Competing combinations are highlighted in pink. Non-competing pairs are shown in blue. **E** Kinetic parameters for the binding of each antibody to CyRPA, as determined by surface plasmon resonance analysis.

Seven new chimeric anti-CyRPA mAbs were produced through immunization of chickens (Cy.003, Cy.004, Cy.007, and Cy.009) and mice (Cy.002, Cy.005, and Cy.010), the former using HybriFree technology^21^ to clone the variable domains of anti-CyRPA IgY mAbs, and the latter using Sp2/0 hybridomas from which the variable domains were sequenced and synthesized. In both cases, the mAb variable domains were subsequently cloned into a hIgG1 backbone. The seven mAbs were initially screened for in vitro growth inhibition activity (GIA) against the 3D7 clone of *P. falciparum* at 0.5 mg/ml (Fig. 1A). Four of the mAbs, Cy.003, Cy.004, Cy.007, and Cy.009, reduced parasite growth by 20% or more, the most potent of these being Cy.004, which showed 81% GIA (Fig. 1A).

To provide a side-by-side comparison of these new antibodies with the most potent published inhibitory anti-CyRPA mAbs, we synthesized chimeric human versions of c12^18^ and 8A7^22^. When compared head-to-head, Cy.004 and Cy.009 inhibited growth with EC_50_ values of 170 and 200 μg/ml respectively (Fig. 1B), while c12 and 8A7 did not achieve 50% inhibition at the top concentration of 2 mg/ml (Fig. 1B). The GIA values reported here for c12 and 8A7 are lower than their published values^18,22^. This can likely be attributed to our use of a one-cycle GIA, as used by the NIH GIA assay reference laboratory^23^, instead of the two-cycle assay used previously for c12 and 8A7. Two-cycle assays have been shown to give higher GIA than one-cycle assays^24^.

We next sorted the antibodies into competition groups, using an ELISA-based assay. Among the four inhibitory mAbs (Cy.003, Cy.004, Cy.007, and Cy.009), only Cy.004 and Cy.009 competed for binding with each other (Fig. 1C, D). All bound conformational epitopes (Supplementary Fig. 1D). Comparison of the amino acid sequences of Cy.004 and Cy.009 revealed CDR loops of equal length with seven amino acid differences contained within heavy chain CDRs and three within the light chain CDRs (Supplementary Fig. 1A). These highly similar sequences, as well as their congruent competition matrices, suggest that these mAbs likely bind to the same epitope. All four inhibitory mAbs competed for binding with 8A7, suggesting that 8A7 binds to the centre of a face containing multiple epitopes for inhibitory antibodies (Fig. 1C, D).

Next, surface plasmon resonance (SPR) was used to measure the kinetics of binding for each mAb to determine if these could explain the differences in GIA. The mAbs bound to CyRPA with affinities ranging from $2.8\times {10}^{-6}$ M for Cy.004 to $7.2\times {10}^{-10}$ M for c12. The two most potent mAbs, Cy.004 and Cy.009, showed the weakest overall affinity in the low micromolar range, due to rapid dissociation rates (>0.1 s^−1^) that approached the limits of the machine (Fig. 1E, Supplementary Fig. 1B, and Supplementary Table 1). Their affinities were, therefore, confirmed using steady-state methods, producing values comparable to those obtained from kinetic fitting ($1.6\times {10}^{-6}\,{{{{{\rm{M}}}}}}$ vs $2.8\times {10}^{-6}\,{{{{{\rm{M}}}}}}$ for Cy.004 and $9.1\times {10}^{-7}\,{{{{{\rm{M}}}}}}$ vs $1.5\times {10}^{-6}\,{{{{{\rm{M}}}}}}$ for Cy.009) (Supplementary Fig. 1C and Supplementary Table 1). In comparison, the less inhibitory mAbs Cy.003 and Cy.007 had higher affinities of $3.8\times {10}^{-8}$ and $1.1\times {10}^{-7}$ M, respectively (Fig. 1E and Supplementary Table 1). The measured affinities for c12 and 8A7 ($1.2\times {10}^{-9}$ M) align with previously reported values^18,25^, indicating that the change to a human IgG1 constant domain did not affect binding affinity. Overall, unlike our observations with antibodies that target RH5^5^, no relationship was observed, in these studies with single antibodies, between GIA and binding parameters, *k*_a_, *k*_d_, or *K*_D_.

### Growth inhibitory mAbs bind blades 1 and 2 of CyRPA

To identify the epitopes for growth-inhibitory monoclonal antibodies, we next crystallized complexes of CyRPA bound to the Fab fragments of Cy.003, Cy.004, and Cy.007. We also crystallized CyRPA in complex with the Fab fragment of non-inhibitory Cy.002. In each case, structures were determined by molecular replacement, using the structure of CyRPA (PDB: 5TIH) and of a Fab fragment with the CDR loops truncated (PDB: 6RCS) as search models.

**Fig. 2:**
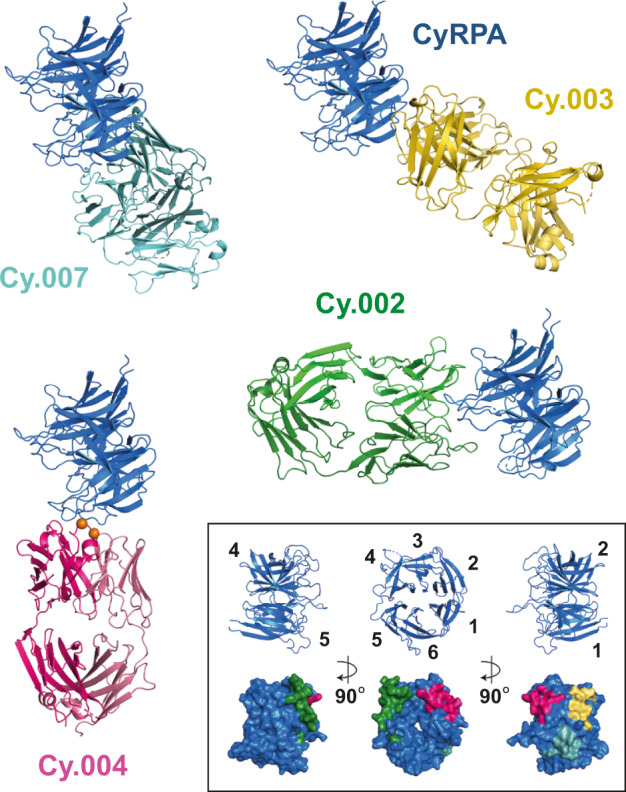
Determining the epitopes for inhibitory and non-inhibitory antibodies targeting CyRPA. Crystal structures of Fab fragments of Cy.002 (green), Cy.003 (yellow), Cy.004 (dark pink), and Cy.007 (light blue), each bound to CyRPA (blue). Within the inset box (bottom right) we show three different views of CyRPA, each rotated by 90°. The upper row “cartoon” images show the six blades of the CyRPA β-propeller. The lower row shows the CyRPA surface (blue) with binding epitopes indicated using the same colours as the antibody structures.

These structures show that all three growth-inhibitory antibodies bind to the same face of CyRPA, with non-overlapping epitopes. CyRPA adopts a β-propeller fold, consisting of six blades^22,26^ and each of the three neutralizing epitopes are largely contained within the first two of these blades. Cy.004 binds predominantly to the second blade, as well as contacting a flexible loop between blades 1 and 2 (Fig. 2 and Supplementary Table 2). Cy.003 also largely binds to blade 2, as well as to a flexible loop between blades 2 and 3 (Fig. 2 and Supplementary Table 2). Cy.007 binding is mediated exclusively by blade 1 (Fig. 2). None of these antibodies bind near the only common polymorphism in CyRPA, R339S^15^.

These data are also consistent with the binding sites of previously identified antibodies c12 and 8A7, which similarly contact blades 1 and 2^22,26^. The c12 epitope largely overlaps with that of Cy.007 (Supplementary Fig. 2), but slight differences in the binding epitope results in clashes with Cy.004, explaining the observed competition (Fig. 1C, D). As expected, superimposition of the 8A7 structure also showed that it binds towards the centre of this key inhibitory face, clashing with c12, Cy.003, Cy.004, and Cy.007 (Supplementary Fig. 2). In contrast, non-inhibitory antibody Cy.002 binds to a different face of CyRPA, contacting blades 3 and 4 (Fig. 2). These structural insights reveal that two blades of CyRPA contain the epitopes for the most growth-inhibitory antibodies.

### Calcium mediates the binding of Cy.004 and Cy.009 to CyRPA

**Fig. 3:**
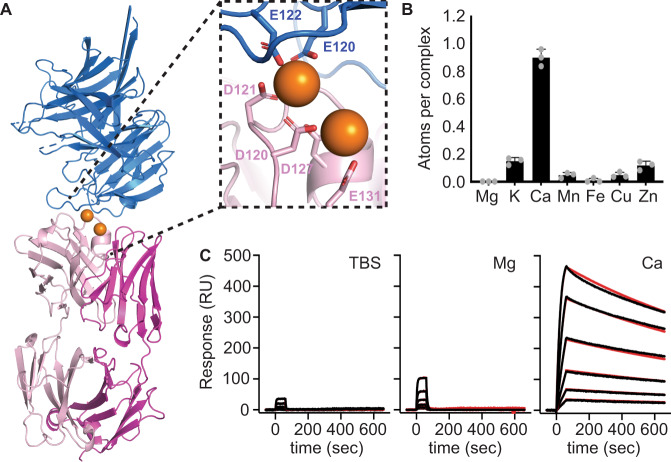
Identification of two calcium ions that mediate the binding of Cy.004 to CyRPA. **A** Cy.004 Fab fragment (pink) bound to CyRPA (blue) with the two calcium ions shown as orange spheres. The inset shows a close up of the calcium-binding site with the coordinating residues labelled. **B** The absolute abundance of different metal ions within the Cy.004:CyRPA complex as determined by microPIXE measurements. Bars indicate the mean and standard deviation across *n* = 3 replicate measurements. **C** SPR sensorgrams of Cy.004 (immobilized) binding to CyRPA in TBS with or without 1 mM MgCl_2_ or 1 mM CaCl_2_. Each curve shows the outcome of a two-fold dilution series of CyRPA from 100 to 3.1 nM. The black line indicates measured response while the red line indicates 1:1 curve fit.

We observed two spheres of electron density within the interface between Cy.004 and CyRPA, whose size and shape were characteristic of metal ions. The first was coordinated by E120 and E122 from CyRPA and D120 and D121 from the heavy chain of Cy.004, while the second was coordinated by D120, D127, and E131, all from the Cy.004 heavy chain (Fig. 3A). The coordinating side chains present “hard” ligands with low polarizability, suggesting divalent cations Mg^2+^ and Ca^2+^ are the ions most likely to bind to these sites^27,28^. To explore this, we used microparticle-induced X-ray emission (microPIXE). Data were collected for the complex of CyRPA bound to Cy.004, purified in lithium acetate buffer to remove ions that would confound the spectra. The most abundant ion identified was calcium, with ~0.9 calcium atoms per complex (Fig. 3B). The same analysis was conducted for the complex of CyRPA and Cy.009, and the predominant ion here was also Ca^2+^ (Supplementary Fig. 3).

As one of the two metal ions mediates interactions between Cy.004 and CyRPA, we measured the affinity of binding using SPR, either in the absence of divalent cations or in the presence of 1 mM CaCl_2_ or 1 mM MgCl_2_. The addition of Ca^2+^ resulted in a >2500-fold decrease in dissociation constant ($1.2\times {10}^{-9}$ M with CaCl_2_ compared to $3.1\times {10}^{-6}$ M without) resulting in comparable affinity to c12 and 8A7. Only a ~13-fold improvement in affinity was seen in the presence of 1 mM MgCl_2_ (2.4 × 10^−7^ M) (Fig. 3C and Supplementary Table 1), supporting the likely identification of the bound ion as Ca^2+^. As calcium is present in serum at approximately 1 mM, which is a component of the parasite growth media for GIA assays, the higher affinity in the presence of Ca^2+^ most likely reflects the physiological affinity. The ubiquitous presence of Ca^2+^ in serum makes it likely that it will be found mediating other antibody–antigen interactions^29^.

### Antibodies Cy.002 and R5.015, that block the interaction between CyRPA and RH5, are non-inhibitory

**Fig. 4:**
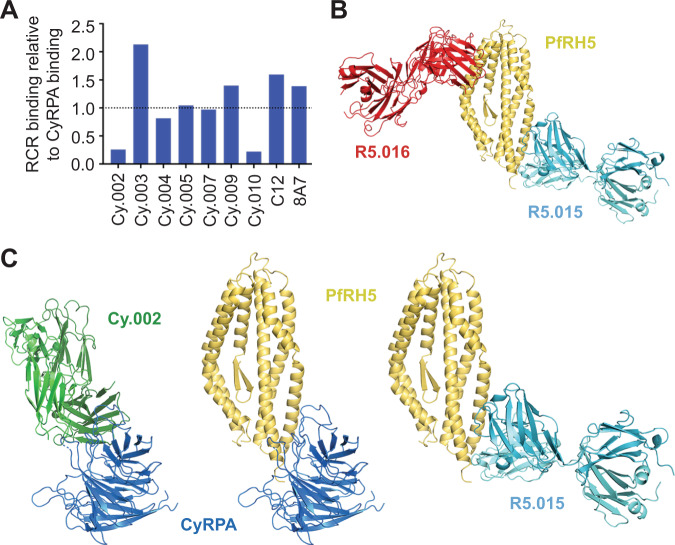
Antibodies which block the RH5:CyRPA interface are not growth inhibitory. **A** Relative binding of 100 nM of each mAb to the reconstituted recombinant RCR complex divided by their binding to CyRPA at the same concentration, as determined by SPR analysis. Dotted line indicates relative binding of 1.0. **B** Crystal structure of RH5 (yellow) bound to Fab fragments of growth inhibitory mAb R5.016 (red) and CyRPA-blocking non-growth inhibitory mAb R5.015 (light blue). **C** Comparison of a model of the CyRPA:RH5 complex, derived from cryo-electron microscopy (PDB: 6MPV), with the structures of CyRPA bound to Cy.002 and RH5 bound to R5.015 shows that both Cy.002 and R5.015 will prevent the formation of the CyRPA:RH5 complex through a steric mechanism.

It has been proposed that antibodies may inhibit erythrocyte invasion by blocking the interaction between CyRPA and RH5, thereby preventing formation of the RCR complex^25^. We, therefore, used SPR to compare the binding of growth inhibitory and non-inhibitory antibodies to CyRPA versus binding to the reconstituted recombinant RCR complex. All of the inhibitory antibodies, including 8A7, which was previously reported to block the RH5:CyRPA interaction^25^, as well as non-inhibitory Cy.005, bound to the assembled RCR complex (Fig. 4A). In contrast, non-inhibitory antibodies Cy.002 and Cy.010 showed markedly reduced binding (relative binding 0.26 and 0.22, respectively) to the RCR complex as compared to CyRPA alone (Fig. 4A). Therefore, neither of the antibodies that bind to epitopes concealed in the RCR complex have neutralizing activity.

To explore this further, we next compared our structure of the CyRPA:Cy.002 complex with a model of the RCR complex derived from cryo-electron microscopy (PDB: 6MPV)^30^. This revealed that CyRPA cannot bind simultaneously to RH5 and to Cy.002 as they share overlapping binding sites, consistent with the low RCR binding measured via SPR (Fig. 4C).

To determine whether a similar conclusion can be derived for antibodies that bind to RH5, and prevent its binding to CyRPA, we studied antibody R5.015^5^. This antibody is not growth inhibitory and also prevents the binding of CyRPA to RH5^5^. We determined a crystal structure of RH5 bound to Fab fragments from both R5.015 and growth inhibitory antibody R5.016. R5.015 binds close to the C-terminus of RH5, through interactions with the C-termini of helices 5 and 7 of RH5, with a buried surface area of 970 Å^2^, while R5.016 binds to the tip of RH5 adjacent to the basigin binding site, as published previously^5^ (Fig. 4B). Comparison with the molecular model of the RCR complex shows R5.015 and CyRPA share overlapping binding sites on RH5^30^ (Fig. 4C).

Together, these two structures indicate that antibodies that bind to either CyRPA or RH5 and that block the RH5:CyRPA interaction are not growth inhibitory. Indeed, all four inhibitory mAbs described here, as well as c12 and 8A7, have epitopes that are accessible in the assembled RCR complex, while Cy.002 and Cy.010 showed reduced binding to the RCR complex and did not inhibit growth. Consistent with these results, none of the eight anti-RH5 mAbs shown to compete with CyRPA for binding have inhibitory activity^5^. While the reasons for this are currently uncertain, one possibility may be that the interaction of RH5 with CyRPA occurs before these proteins become exposed to antibodies at the parasite surface, thereby occluding the epitopes for antibodies such as Cy.002 and R5.015. Alternatively, it may be that the formation of this complex is not essential for erythrocyte invasion. In either case, these data make clear that vaccines to prevent blood-stage malaria should not aim to induce antibodies that block the interaction between RH5 and CyRPA.

### Synergistic GIA occurs with pairs of inhibitory anti-CyRPA antibodies

To understand the efficacy of different combinations of anti-CyRPA mAbs, a comprehensive pair-wise synergy analysis was undertaken using the Bliss definition of additivity^31^, as reported previously^32^. Here, all four newly described inhibitory anti-CyRPA mAbs, as well as 8A7, were assessed for GIA in pair-wise combinations. In each assay, one mAb was held at a constant concentration to yield approximately 20% GIA, while a second mAb was titrated in using a seven-step four-fold dilution curve, starting at 2 mg/ml, as described previously^5,7,33^.

**Fig. 5:**
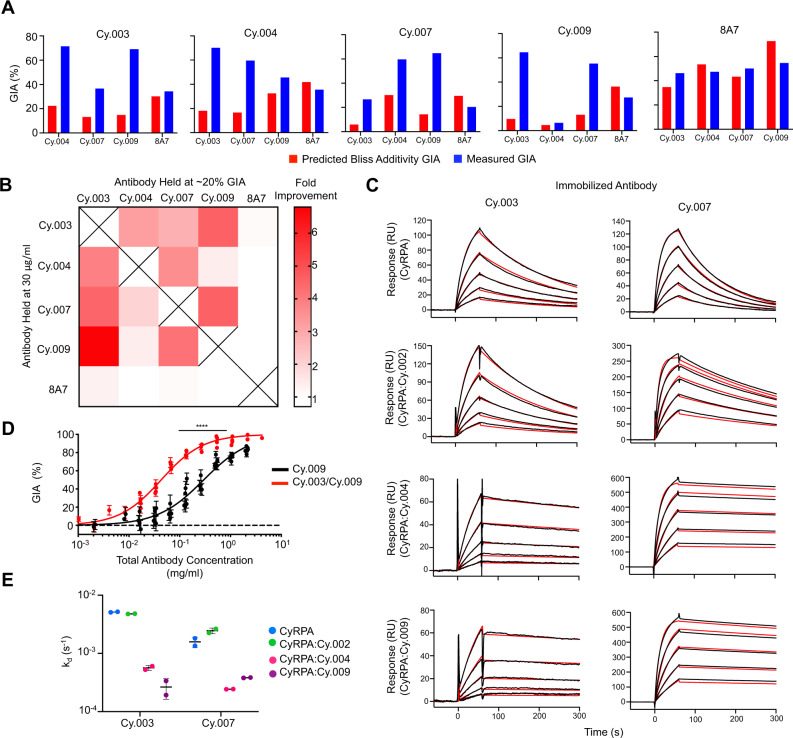
Demonstration of synergistic growth inhibitory activity of monoclonal antibodies targeting CyRPA. **A** Predicted growth inhibitory activity (GIA) based on Bliss additivity (red) compared to measured GIA in blue for a mAb combination where one was held at 30 μg/ml (title) and the other held at 20% GIA (*X*-axis). Complete dilution curves can be seen in Supplementary Fig. 4A. Combinations were measured twice with a single representative experiment shown here. Bar indicates the mean across triplicate measurements. **B** Heat map summary of the fold improvement over Bliss additivity from panel (**A**). **C** SPR sensorgrams of Cy.003 and Cy.007 binding to CyRPA and Fab:CyRPA complexes of Cy.002, Cy.004, or Cy.009 in TBS with 1 mM CaCl_2_. The black lines indicate the measured response while red lines indicate the curve fit. Each graph shows a five-step two-fold dilution curve starting from 500 nM. **D** GIA of Cy.009 compared to that of a Cy.003/Cy.009 1:1 mixture (i.e., 1 mg/ml = 1 mg/ml Cy.009 or 0.5 mg/ml Cy.009 + 0.5 mg/ml Cy.003). Each individual point is the mean of a triplicate measurement with error bars indicating the standard deviation. *n* = 5 and *n* = 6 independent experiments of complete dilution curves for Cy.009 and Cy.003/Cy.009 respectively. Curve fit used a four-parameter dose-response curve with the upper bound constrained to 100% GIA. EC_50_ shift determined through extra-sum-of-squares F test (*F* = 77.68, DFn = 1, DFd = 80), *p* < 0.0001. **E** Summary of changes in dissociation rate constant (*k*_d_) for SPR data shown in (**C**). Horizontal line indicates the mean and error bars show the standard deviation across *n* = 2 independent experiments.

As expected, 8A7, which competes for CyRPA binding with all the other inhibitory mAbs, did not synergize with them, instead showing Bliss additivity or weakly antagonistic behaviour (Fig. 5A, B). This is particularly evident when inhibition across the entire dilution curve is examined (Supplementary Fig. 4A). Likewise, no improvement in GIA (above the level due to the inhibitory mAb alone) was seen when any of the inhibitory mAbs were used in combination with non-inhibitory mAb Cy.002 (Supplementary Fig. 4B).

In contrast, synergy was observed when Cy.003, Cy.004, Cy.007, and Cy.009 were used in combination (Fig. 5A, B and Supplementary Fig. 4A). All pair-wise combinations of these four antibodies showed GIA at least two-fold above predicted Bliss additivity, except for Cy.004 and Cy.009, which is consistent with them binding the same epitope. As high as six-fold improvement over Bliss additivity was observed in the case of Cy.003 with Cy.009. These results were consistent for each antibody pair regardless of which mAb in the pair was held constant and which was titrated (Fig. 5A, B and Supplementary Fig. 4A).

To confirm this observation, the most potent combination of two mAbs, Cy.003 and Cy.009, was prepared in a 1:1 ratio and compared with Cy.009 alone at equal concentrations. Due to the reduced activity of Cy.003 compared to Cy.009, one would expect the performance of the combination to be worse than Cy.009 if there is no interaction between the two mAbs. Instead, a decrease in EC_50_ from 297 μg/ml (95% CI: 272–325 μg/ml) to 43 μg/ml (95% CI: 40–46 μg/ml) was seen (*p* < 0.0001) (Fig. 5D). This improvement in EC_50_, driven by antibody synergy, makes this anti-CyRPA mAb combination comparable to some of the more potent anti-RH5 clones^5,34^.

### Synergy occurs due to improved binding kinetics as a result of lateral antibody–antibody interactions

We next investigated the molecular mechanisms underlying antibody synergy. Synergy due to an allosteric mechanism was ruled out since structural studies show that binding by Cy.003, Cy.004, and Cy.007 caused no conformational changes in CyRPA (Fig. 2). We, therefore, explored whether synergy might result from changes in binding kinetics.

SPR was used to determine the change in binding kinetics of a lower affinity mAb when a higher affinity synergistic mAb was present. In the presence of 1 mM CaCl_2_, the *k*_d_ of Cy.003 was reduced from $5.1\times {10}^{-3}\,{{{{{{\rm{s}}}}}}}^{-1}$ for CyRPA alone to $5.7\times {10}^{-4}\,{{{{{{\rm{s}}}}}}}^{-1}$ in the presence of Cy.004 and to $2.7\times {10}^{-4}\,{{{{{{\rm{s}}}}}}}^{-1}$ in the presence of Cy.009 (Fig. 5C, E). Comparable decreases were observed for Cy.007, with a *k*_d_ of $1.6\times {10}^{-3}\,{{{{{{\rm{s}}}}}}}^{-1}$ for CyRPA alone compared to $2.4\times {10}^{-4}\,{{{{{{\rm{s}}}}}}}^{-1}$ and $3.8\times {10}^{-4}\,{{{{{{\rm{s}}}}}}}^{-1}$ in the presence of Cy.004 and Cy.009 respectively (Fig. 5C, E). Meanwhile, the non-synergistic mAb Cy.002 had no impact on dissociation. Despite no improvement in the rate of association in the presence of a synergistic Fab, the reductions in the *k*_d_ resulted in a higher overall affinity in all synergistic pairs compared to CyRPA alone (Supplementary Fig. 4C). These results were also confirmed in the absence of calcium. Without CaCl_2_ in the buffer, both Cy.004 and Cy.009 showed rapid dissociation, with *k*_d_ values of 0.49 and $0.14\,{{{{{{\rm{s}}}}}}}^{-1}$ respectively (Supplementary Fig. 4D, E and Supplementary Table 1). This dissociation dramatically slowed when the analyte used was CyRPA bound to Cy.003 or Cy.007 Fab fragments (Supplementary Fig. 4D, E and Supplementary Table 1). For Cy.004, the *k*_d_ values for binding to Cy.003:CyRPA and Cy.007:CyRPA were $5.9\times {10}^{-2}$ and $1.5\times {10}^{-2}\,{{{{{{\rm{s}}}}}}}^{-1}$, respectively. For Cy.009, the *k*_d_ values for binding to Cy.003:CyRPA and Cy.007:CyRPA were $1.3\times {10}^{-2}$ and $2.7\times {10}^{-2}\,{{{{{{\rm{s}}}}}}}^{-1}$, respectively. Therefore, in each case, antibody synergy is associated with stronger binding to CyRPA and slower dissociation of the antibodies from CyRPA.

To determine the molecular basis for improved binding kinetics, we determined a crystal structure of CyRPA in complex with the Fab fragments of Cy.003, Cy.004, and Cy.007. This crystallized with four copies in the asymmetric unit and the structure was determined to 3.3 Å resolution using molecular replacement with the individual structures obtained earlier used as molecular replacement search models. The conformation of CyRPA and the location and nature of the epitopes for the three antibodies were largely unchanged from those observed in structures of CyRPA bound to single antibodies, with the exception of Cy.004, which showed a small rotation.

**Fig. 6:**
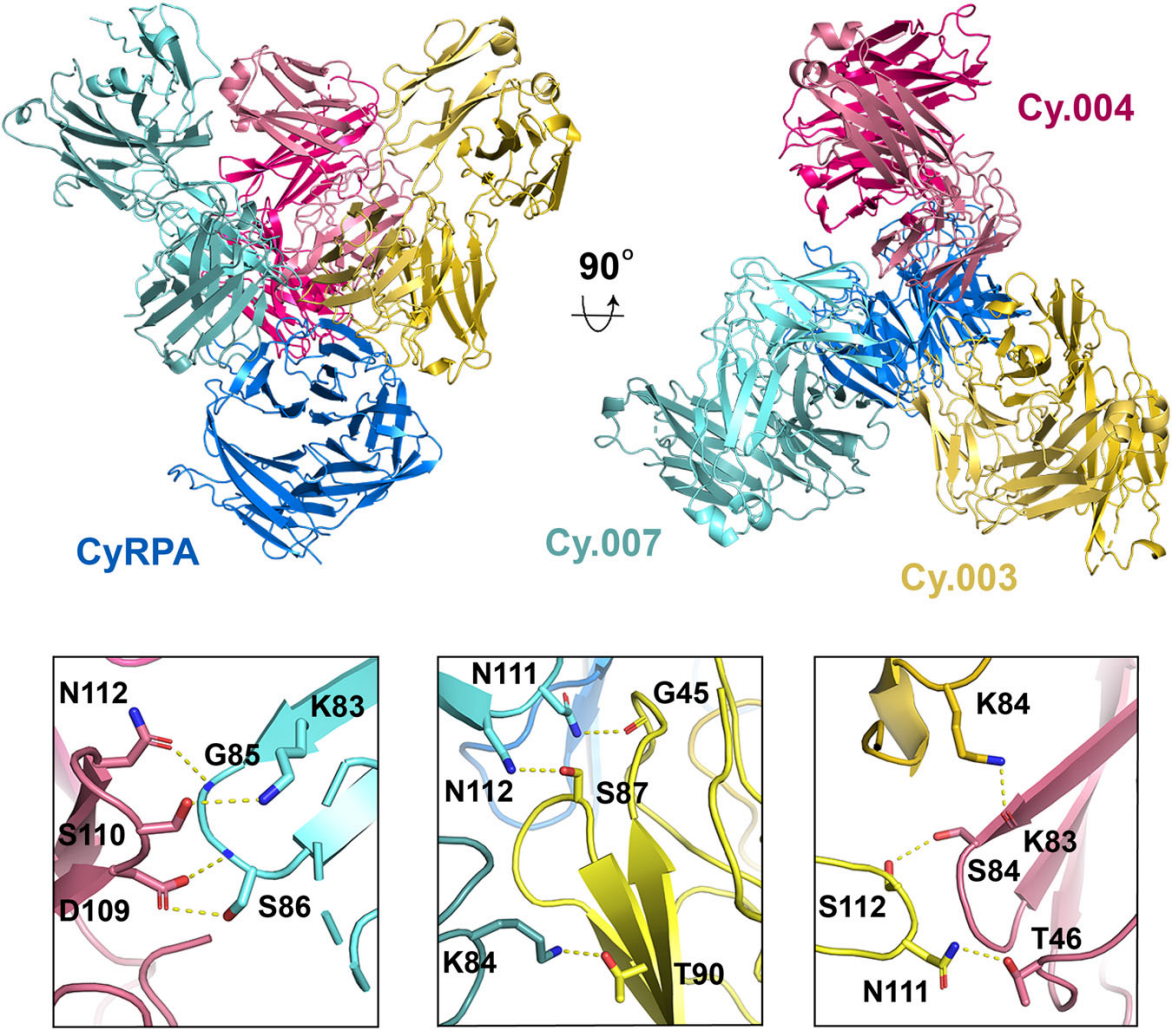
Synergy between different antibody pairs is mediated through lateral heterotypic interactions. Crystal structure of CyRPA (blue) bound to Fab fragments of Cy.003 (yellow), Cy.004 (pink), and Cy.007 (light blue). The lower panels show close-up views of each of the interfaces between Cy.004 and Cy.007 (left), Cy.003 and Cy.007 (centre), and Cy.004 and Cy.003 (right). The residues forming heterotypic interactions are labelled and bonds are indicated with yellow dashed lines.

The most striking observation from this structure was the presence of lateral heterotypic Fab–Fab interactions between each of the three antibodies (Fig. 6 and Supplementary Table 3). Based on this structure, hydrogen bonding via side chain-side chain and side chain-main chain interactions likely stabilize antibody binding, thereby slowing dissociation (Fig. 6). CDR loops from neighbouring mAbs play a critical role in these interactions, with both CDR:CDR interactions (LCDR1 of Cy.003 to LCDR3 of Cy.007, and LCDR1 of Cy.004 to LCDR3 of Cy.003), as well as CDR interactions with framework (LCDR3 of Cy.007 to Cy.003 framework and LCDR3 of Cy.004 to Cy.007 framework) forming these interacting interfaces (Fig. 6). Lateral interactions also occurred between framework regions, with both heavy and light chain frameworks of Cy.003 interacting with the frameworks of Cy.004 and Cy.007 light chains (Supplementary Table 3).

## Discussion

In this study, we describe our detailed characterization of a panel of monoclonal antibodies targeting the essential and highly conserved blood-stage malaria vaccine candidate antigen CyRPA, with the aim of using the insight gained to guide the design of improved CyRPA-based vaccine immunogens. Firstly, we reveal the region of CyRPA that contains the epitopes for the most neutralizing antibodies, and we show that antibodies that interrupt the interaction between CyRPA and RH5, by targeting either antigen, are not effective at blocking parasite growth. Instead, antibodies that target blades 1 and 2 of the β-propeller of CyRPA are the most growth inhibitory. This surface was previously shown to contain the epitopes of neutralizing antibodies c12^26^ and 8A7^22^ and it is also targeted by all three of the most growth-inhibitory antibodies identified in this study.

The role of CyRPA in erythrocyte invasion by the merozoite is still unknown, making it currently impossible to rationalize the mechanism behind growth inhibition or to explain the differences in potency between different antibodies. Indeed, it is uncertain why Cy.004 and Cy.009 are substantially more inhibitory than the other antibodies published to date^18,22^, or than other antibodies identified in this study. The binding of Cy.004 and Cy.009 to CyRPA is not stronger or faster than that of the other neutralizing antibodies, based on binding kinetics. This suggests that improved potency is mostly likely due to the specific location of the Cy.004/Cy.009 epitope and that future insight into the role of CyRPA during invasion will be required before this can be understood. However, these data demonstrate that the most effective neutralizing antibodies can bind to CyRPA when it is present within the formed RCR complex. Indeed, it is the face formed from blades 1 and 2 of CyRPA that appears most exposed when CyRPA is assembled into the RCR^30^. Future vaccine immunogens based on CyRPA should display this surface.

The most striking finding from this study is our identification of lateral heterotypic Fab–Fab interactions between antibodies that bind to neighbouring epitopes. We also show that pairs of antibodies that make such lateral interactions synergize with one another, with both improved stability of their complex with CyRPA and improved GIA. While antibody–antibody interactions have been previously seen for multiple copies of the same antibody that bind to identical epitopes on a repeating antigen^10–12^ or a multimeric antigen^13^, we are not aware of another demonstration of synergy through heterotypic interactions. Nevertheless, it seems likely that this mechanism will be found repeatedly as larger antibody panels are studied. Indeed, as the majority of the lateral interactions between CyRPA-targeting antibodies are mediated by CDR loops, their formation might be expected to result from normal processes of antibody diversification.

Whether such heterotypic interactions can be selected for during evolution of the adaptive immune response in humans remains an important question. It was previously shown that homotypic interactions can enhance B cell activation and can be selected through affinity maturation of framework residues^10^. A similar phenomenon could occur in the evolution of heterotypic interactions, wherein a secreted antibody, coupled to antigen in immune complexes displayed within the germinal centre, may interact with B cell receptors bound to adjacent epitopes, slowing antigen dissociation, enhancing binding affinity, and increasing B cell activation. This may favour the amplification of those cells that express antibodies capable of forming heterotypic interactions. Importantly, the improvements in affinity as a result of dual antibody binding (in the presence of calcium) are from ~10^−7^ to ~10^−9^ M, which is within the range of discrimination of BCRs^35^, indicating that these interactions could be selected for during antibody maturation.

The discovery of lateral heterotypic interactions between antibodies also has consequences for rational vaccine design. One of the achievements of structure-guided immunogen design is the ability to generate immunogens capable of eliciting an epitope-focused antibody response^36,37^. Immunogens can be produced either by grafting a particular epitope onto a scaffold protein^36^, or through de novo design of a protein that presents a single epitope^37^. Such immunogens specifically elicit antibodies that target a single epitope and have been used in cases in which neutralizing antibodies are challenging to elicit. Recently, this approach has been extended to the design of three separate immunogens, each of which induces antibodies against a different epitope^37^. This study of synergistic CyRPA-targeting mAbs highlights a potential risk of such an approach as, in some cases, each immunogen might present an epitope region too small to allow the induction of a polyclonal response in which different antibodies can synergize through heterotypic interactions. In the case of CyRPA, a better outcome is predicted from an immunogen presenting all of blades 1 and 2, thereby allowing induction of antibodies that mediate lateral interactions to combine to generate a sufficiently growth-inhibitory polyclonal response.

Beyond vaccination, there is growing interest in the deployment of monoclonal antibodies against infectious disease both prophylactically and therapeutically^38^. In the context of malaria, a recent report has shown the promise of this approach against the liver stage of infection, where all nine recipients of the CIS43LS mAb were protected in a controlled human malaria infection challenge study^39^. Equivalent studies have not yet commenced for blood-stage vaccine antigens, but likely will in the future. Promoting lateral interactions that enhance biological activity may then prove to be an important component of cocktail design. Indeed, it may be that a mechanism to favour the formation of such a synergistic antibody mixture could be to select or induce antibodies through immunization with a complex consisting of an antigen complexed to a monoclonal antibody. Such an approach has the potential to generate antibody cocktails which, through synergy, are greater than the sum of their constituents.

With antibody panels against vaccine candidates now including hundreds of unique clones^40^ and methods for structural assessment of polyclonal antibody responses improving^41^, we expect more examples to emerge of antibody synergy driven by heterotypic lateral interactions. Mapping of the intricate ways in which antibodies interact to enhance protection will then allow the design of vaccine immunogens that enable the generation of synergistic antibody responses to tackle challenging diseases.

## Methods

### Microbe strains

Parasite experiments were carried out using the 3D7 clone of *P. falciparum*. Parasites were cultured in unvented flasks using complete culture medium (RPMI, 10% heat-inactivated human serum, 20 μg/ml gentamycin, 2 mM L-glutamine, 0.05 g/L hypoxanthine, 5.94 g/L HEPES) at 37 °C with 2 % hematocrit using O + red blood cells in a 5% O_2_, 5% CO_2_, 90% N_2_ atmosphere.

### Cell lines

Expi293F cells (Thermo Fisher Scientific) were maintained in suspension in Expi293 expression medium (Thermo Fisher Scientific) at 37 °C, 8% CO_2_, in a shaking incubator at 120 rpm.

*Drosophila* S2 cells^42^ were cultured at 25 °C using EX-CELL 420 medium (Sigma-Aldrich) with 100 U/ml penicillin, 0.1 mg/ml streptomycin, and 10% fetal bovine serum at 115 rpm in an Innova 44 shaking incubator.

### Antibody generation

c12^18^ and 8A7^22^ were previously published mouse-derived antibodies. Here, we synthesized the nucleotide sequence of the light and heavy variable domains, based on published amino acid sequences (PDB: 5TIH, 5EZO) (GeneArt). The heavy and light chains were then cloned into AbVec-hIgG1/AbVec-hIgG1-kappa vectors^43^, respectively, through digestion of both the synthesized vector and destination vector with AgeI/SalI (New England Biolabs) and AgeI/BsiWI (New England Biolabs), respectively, for 60 min at 37 °C followed by overnight ligation at 16 °C. Ligated vectors were then transformed into DH5α competent *Escherichia coli* (New England Biolabs), streaked onto LB agar plates with 100 μg/ml carbenicillin, and incubated overnight at 37 °C.

One panel of mAbs (Cy.003, Cy.004, Cy.007, and Cy.009) were produced by Icosagen using HybriFree Technology, as described previously^21^. For clarity, these mAbs were renamed from their original EURIPRED consortium catalogue names, which should be cited for reagent requests (Cy.003 = 3B3#17, Cy.004 = 4D12#30, Cy.007 = 3A7#22, Cy.009 = 7B9#13) (Nacer et al. manuscript in preparation). Two chickens were immunized with 0.5 mg of recombinant CyRPA in complete (first immunization) or incomplete Freund’s adjuvant (all other doses) at 0, 2, 4 and 18 weeks via intramuscular injection, followed by an intravenous injection of 0.1 mg CyRPA in PBS at week 20. Antigen-coated (5 μg/mL) immune modules (Thermo Fisher Scientific) were used to pan 2 × 10^4^ spleen cells from immunized animals. Unbound cells were removed by washes in phosphate-buffered saline (PBS) following a 45 min incubation. RNA was extracted from the bound cells and used to synthesize cDNA using Superscript IV First-Strand Synthesis System (Invitrogen) for reverse transcription polymerase chain reaction (RT-PCR) and amplification of the variable light (VL) and heavy (VH) chains. The amplified VL and VH were purified and cloned into human immunoglobulin G1 (hIgG1) expression vectors by circular polymerase extension cloning (CPEC). Antibody sequences are in Supplementary Table 5.

A second set of mAbs (Cy.002, Cy.005, Cy.010) were produced through immunization of 6–8 week old female BALB/c mice (Harlan Laboratories) with 20 μg CyRPA in PBS and mixed with 50μL Addavax^TM^ (Invivogen) following an 8-week prime-boost schedule, as previously described^34^. Briefly, spleens were harvested 3 days post-boost, and splenocytes were fused with Sp2/0 myeloma cells (ECACC) before plating in methylcellulose-based medium (ClonacellHY). Hybridomas were then screened for CyRPA binding via ELISA. After preliminary characterization of CyRPA binding, the variable domains of the heavy and light chains were sequenced, synthesized, and cloned into AbVec-hIgG1/AbVec-hIgG1-kappa vectors respectively^43^ following the same procedure as described above for c12 and 8A7. All procedures on mice were performed in accordance with the terms of the UK Animals (Scientific Procedures) Act Project Licence (PA7D20B85) and were approved by the University of Oxford Animal Welfare and Ethical Review Body. The mice were housed in individually ventilated cages at 20–24 °C with 12 h light and dark cycles.

### Protein expression and purification

#### CyRPA

The CyRPA construct used was based on the CyRPA sequence from the 3D7 clone of *P. falciparum* encompassing amino acids 29–362. A mammalian secretion peptide, MEFQTQVLMSLLLCMSGAAA, was added upstream of residue 29 to enable secretion from a mammalian expression system, along with a four-glycine linker followed by an EPEA tag (C-tag) on the C-terminus^44^. The following mutations were introduced to remove three potential N-linked glycosylation sites: S147A, T324A, and T340A.

HEK Expi293 cells (Thermo Fisher Scientific) were transfected following the manufacturer’s protocol using expifectamine (Thermo Fisher Scientific), including the addition of enhancer 1 and enhancer 2 (Thermo Fisher Scientific) 18 h post-transfection. Supernatants were harvested 4 days after initial transfection via centrifugation.

CyRPA was first purified through C-tag affinity purification using a 10 ml column packed with CaptureSelect C-tag affinity resin (Thermo Fisher Scientific). Harvested supernatants were run over the column at 5 ml/min and then washed for 10 column volumes (CV) with TBS (150 mM NaCl, 20 mM Tris pH 7.4); CyRPA was eluted using 2 M MgCl_2_. Fractions were pooled and concentrated to 2 ml before size exclusion chromatography (SEC) using an S200 16/600 column (GE Healthcare) on an Äkta pure (GE Healthcare) into TBS.

#### Antibodies

mAbs were produced via transient transfection of HEK Expi293 cells (Thermo Fisher Scientific) following the same procedure as described above for CyRPA. mAb was purified from culture supernatants via protein G purification using a 5 ml protein G column (GE Healthcare) in TBS. mAb was eluted in 1.6 ml fractions in glycine (200 mM, pH 2.0) into Tris buffer (1 M, pH 9.0). Fractions were pooled and concentrated to 2 ml before SEC using an S200 16/600 column (GE Healthcare). Fabs were produced through papain digestion using the Fab Preparation kit (Thermo Fisher Scientific) following the manufacturer’s instructions.

#### RH5

Production of full-length recombinant RH5 in *Drosophila* S2 cells was as previously published^42^. The RH5 sequence was based on the 3D7 clone of *P. falciparum* with four mutations to remove putative glycosylation sites (T40A, T216A, T286A, and T299A) and a C-terminal C-tag (EPEA). Cells were harvested in 2 L batches and then subjected to tangential flow filtration (Millipore Pellicon 3) with a 10 kDa cut-off. Subsequently, RH5 was purified using the same two-step purification as CyRPA: affinity purification using a 10 ml CaptureSelect C-tag affinity resin (Thermo Fisher Scientific) and SEC using an S200 16/600 column and Äkta pure (GE Healthcare) in into TBS.

#### RIPR

RIPR (amino acids 21–1086) was expressed in *Drosophila* S2 cells and subject to the same purification process as RH5 (C-tag affinity chromatography followed by SEC into TBS). Eight residues were mutated to eliminate putative glycosylation sites (N103Q, N144Q, N334Q, N480Q, N498Q, N506Q, N526Q, N646Q, N964Q, and N1021).

#### RCR complex

After purification of RH5, CyRPA and RIPR, assembled RCR complex was produced by mixing equimolar concentrations of each protein and incubating for 60 min at room temperature. The assembled complex was then purified by SEC using an S200 16/600 column and Äkta pure (GE Healthcare) into TBS.

For storage, all protein samples were flash-frozen in liquid nitrogen in 1 ml aliquots.

### Surface plasmon resonance

#### Antibody kinetics

Purified mAb was immobilized on a protein G chip through a 30 s injection of 20 nM mAb. CyRPA was diluted in PBS + P20 running buffer (137 mM NaCl, 2.7 mM KCl, 10 mM Na_2_HPO_4_, 1.8 mM KH_2_PO_4_, 0.005% surfactant P20 (GE Healthcare)) to yield a top concentration of 125–500 nM (depending on antibody affinity and required concentration range). Samples were injected for 60 s at 30 μL/min before dissociation for 600 s. The chip was then regenerated with a 45 s injection of 10 mM glycine pH 1.5. Antibody kinetics were determined through a two-fold, six-step dilution curve. Data were analyzed using the Biacore X100 Evaluation sample. A global Langmuir 1:1 interaction model was used to determine antibody kinetics.

For kinetic analyses in the presence of calcium or magnesium, the same procedure was used starting from a top concentration of 100 nM, using TBS + P20 (150 mM NaCl, 20 mM Tris pH 7.4, 0.005% surfactant P20) with or without 1 mM CaCl_2_ or MgCl_2_. TBS was used in place of PBS due to the presence of visible precipitate for PBS with 1 mM CaCl_2_.

#### Steady state affinity

For mAbs with rapid dissociation (Cy.004 and Cy.009), mAb binding affinity was also determined through steady-state analysis to ensure measurement accuracy due to *k*_d_ measurements approaching or exceeding the machine limits. 300 RU of Cy.004 or Cy.009 were immobilized on a CM5 chip (GE Healthcare) using an amine coupling kit (GE Healthcare) in 10 mM sodium acetate pH 5. Analytes in PBS + P20 were injected for 60 s at 30 μL/min before dissociation for 250 s. The chip was then regenerated with a 45 s injection of 10 mM glycine pH 2. Steady state affinity was determined across a seven-step, two-fold dilution curve beginning at 4 μM.

#### Dual binding kinetics

300 RU of the lower affinity mAb was immobilized to a CM5 chip using an amine coupling kit (GE Healthcare) as above. Purified Fab fragment from the higher affinity mAb in the pair was incubated in equimolar concentrations with CyRPA at room temperature for 30 min and then purified on an S200 10/300 column (GE Healthcare). Analytes were injected for 60 s at 30 μL/min before dissociation for 250 s for a five-step, two-fold dilution curve starting at 500 nM. The chip was then regenerated with a 45 s injection of 10 mM glycine pH 2.

#### RCR binding

A mAb of interest was immobilized on a protein G chip through a 30 s injection of a 20 nM solution of mAb in PBS + P20. 100 nM CyRPA alone was injected for 45 s before allowing for 250 s of dissociation. The protein G chip was regenerated through a 45 s injection with 10 mM glycine pH 1.5 followed by injection of 100 nM of the RCR complex, following the same procedure. Relative binding of RCR was calculated as Peak Response (RCR)/Peak Response (CyRPA).

### ELISA

#### CyRPA binding

96-well NUNC immunoplates (Thermo Fisher Scientific) were coated with 50 μL of either native state or denatured (boiled at 95 °C for 5 min and reduced using 5 mM dithiothreitol) CyRPA at 2 μg/ml overnight at 4 °C. The plates were then washed with PBS/Tween-20 (PBS-T) (137 mM NaCl, 2.7 mM KCl, 10 mM Na_2_HPO_4_, 1.8 mM KH_2_PO_4_, 0.05 % Tween-20) three times. Next, plates were blocked with PBS (137 mM NaCl, 2.7 mM KCl, 10 mM Na_2_HPO_4_, 1.8 mM KH_2_PO_4_) with 1% casein for 60 min at room temperature. Plates were then washed with PBS-T before the addition of 50 μL of mAb at 10 μg/ml, diluted in PBS-casein. mAb solution was incubated for 60 min at room temperature before washing with PBS-T. Next, 50 μL of goat anti-human whole IgG alkaline phosphate conjugate (Sigma-Aldrich), diluted 1/1000 in PBS with 1% casein, was added to each well and then incubated for 60 min at room temperature, then washed. Next, a 20 mg tablet of 4-Nitrophenyl phosphate (pNPP, Sigma-Aldrich) was dissolved in 4 ml of diethanolamine buffer (Thermo Fisher Scientific) with 16 ml deionized water. 100 μL pNPP was then added to each well and plates were allowed to develop until the positive control (mAb c12) reached an OD_405_ of approximately 2.0. The anti-RH5 mAb, R5.011^5^, was used as a negative control as it should not bind to CyRPA.

#### Epitope binning

96-well NUNC immune plates (Thermo Fisher Scientific) were coated with 50 μL mAb at 2 μg/ml in PBS overnight at 4 °C. The following day, each well was blocked with 100 μL of PBS with 1% casein (Pierce) for 1 h at room temperature. After blocking, the plate was washed with PBS-T. Next, 50 μL CyRPA was added to each well at a concentration of 10 μg/ml in PBS with 1% casein. After 60 min, the well contents were discarded and then washed 3× with PBS-T. Next, 50 μL of the “competing” anti-CyRPA biotinylated mAb (biotinylated using antibody biotinylation kit, according to the manufacturer’s instructions (Thermo Fisher Scientific)) at 10 μg/ml was added to each well and incubated for 60 min, followed by 3× wash steps with PBS-T. 50 μL streptavidin-alkaline phosphatase (Thermo Fisher Scientific) diluted 1/1000 in PBS-casein, was then added, incubated for 15 min, and washed 6× with PBS-T. Detection using pNPP was done as described above. Biotinylated antibody binding to CyRPA coated directly onto the plate was used as the positive control, while self-competition (same capture and detection antibody) was used as the negative control.

### Assay of growth inhibition activity

mAbs were buffer exchanged into incomplete parasite growth media (RPMI, 2 mM L-glutamine, 0.05 g/L hypoxanthine, 5.94 g/L HEPES) before performing a one-cycle GIA, as previously published^23^. To ensure consistency between experiments, in each case the activity of a negative control mAb, EBL04^45^, which binds to the Ebola virus glycoprotein, and three anti-RH5 mAbs with known GIA^34^ (2AC7, QA5, 9AD4) were run alongside the test mAbs. For the synergy GIA, each pair of mAbs was assessed by measuring GIA of: (1) one mAb was held constant at approximately 20 % GIA, (Cy.003 = 0.7 mg/ml, Cy.004 = 0.2 mg/ml, Cy.007 = 2 mg/ml, Cy.009 = 0.2 mg/ml, 8A7 = 2 mg/ml); (2) a second mAb across a four-fold seven-step dilution curve beginning at 2 mg/ml; and (3) the combination of the first mAb held at a constant concentration with the second mAb across its dilution curve. The Bliss additivity^31^ was determined based on the measured activity from each antibody alone (1 and 2) using the following formula:

1 $${{{{{{\rm{GIA}}}}}}}_{1+2}=\left[1-\left(1-\frac{{{{{{{\rm{GIA}}}}}}}_{1}}{100}\right)\times \left(1-\frac{{{{{{{\rm{GIA}}}}}}}_{2}}{100}\right)\right]\times 100$$

### MicroPIXE

Complexes were made by mixing 0.2 mg of CyRPA and 0.35 mg of mAb. MgSO_4_ and CaCl_2_ were each added to 1 mM final concentration and the samples were incubated for 30 min. The sample was then run through an S200 10/300 column, pre-equilibrated in a buffer consisting of 100 mM lithium acetate titrated to pH 7.0 using 100 mM boric acid. The measurements were carried out at the Ion Beam Centre, University of Surrey, UK. A 350 pA 2.5 MeV proton beam of diameter ~2.0 µm was used to induce characteristic X-ray emission from dried protein complex droplets (volume per droplet ~ 0.1 µl) under vacuum. The X-rays were detected using a silicon drift detector (Rayspec Ltd, U.K.) with active area 80 mm^2^ and energy resolution of 130 eV at 5.9 keV. By scanning the proton beam in *x* and *y* over the dried samples, spatial maps were obtained of all elements heavier than neon present in the sample. Quantitative information, using sulfur as an internal standard, was obtained by collecting 3 or 4 point spectra from each droplet. These spectra were analyzed with GUPIX^46^ within DAN32^47^ to extract the relative amount of each element, particularly calcium, in the sample. Comparison of the quantities of sulfur and calcium allowed the determination of the number of calcium ions per protein complex.

### Crystallography

Fab(s) and CyRPA were mixed in equimolar ratios to yield a total of 4 mg of protein complex and incubated at room temperature for 30 min in low salt TBS (50 mM NaCl, 20 mM Tris pH 8) before purification on a S200 16/600 column using an Äkta pure (GE Healthcare). Fractions containing assembled complex were pooled and concentrated to approximately 10 mg/ml using an Amicon ultra centrifugal filters (Millipore) with a 50 kDa molecular weight cut-off.

mAb:CyRPA crystallization screens were conducted using a sitting drop vapour diffusion approach. 200 nL drops composed of 50% of protein solution and 50% of crystallization condition were prepared using a Mosquito low volume nL pipetting instrument (TTP Labtech). All complexes were screened against the JCSG+, Proplex, PACT, Midas, and Morpheus screens (Molecular Dimensions) at two temperatures, 4 and 18 °C. Cy.003:Cy.004:Cy.007:CyRPA and R5.015:R5.016:CyRPA underwent additional optimization using the silver bullets additive screen where 50 nL of additive was added per drop.

All diffracting crystals grew at room temperature. The crystallization conditions that yielded diffracting crystals for each complex were: 0.2 M NaCl, 0.1 M MES pH 6, 20% PEG 2000 MME (Cy.003:CyRPA); 20% PEG 6000, 0.1 M Tris pH 8.5 (Cy.004:CyRPA); 0.2 M zinc acetate, 10% PEG 3000, 0.1 M sodium acetate pH 4.5 (Cy.007:CyRPA); 0.1 M lithium sulfate, 30% polyvinylpyrrolidone K15, 0.1 M HEPES pH7 (Cy.002:CyRPA); 0.09 M MD Morpheus Halogens Mix, 30% EDO_P8K, 0.1 MMD Morpheus Buffer System 1 pH 6.5 with an additive containing 0.25% 1,5-Naphthalenedisulfonic acid disodium salt, 0.25% 2,7-naphthalenedisulfonic acid disodium salt, 0.25% 5-Sulfoisophthalic acid monosodium salt, 0.25% sulfanilic acid, 0.02 M HEPES sodium pH 6 (Cy.003:Cy.004:Cy.007:CyRPA); and 0.2 M sodium formate, 0.1 M bis-tris propane pH 6.5, 20% PEG 3350 with an additive containing 0.08% w/v Ellipticine, 0.20% w/v Protamine sulfate salt, 0.20% w/v D-(+)-Trehalose dihydrate, 0.20% w/v 6-Phosphogluconic acid trisodium salt, 0.20% w/v D-(+)-Glucose, 0.02 M HEPES sodium pH 6.8 (R5.015:R5.016:RH5). If they were not already cryoprotected, crystals were transferred into a 2 μL drop of mother liquid containing cryoprotectant (25% glycerol) before flash-freezing in liquid nitrogen.

Data were collected using a wavelength between 0.95 and 1 Å from the Diamond Light Source on beamline I03 (Cy.003:CyRPA), I04 (Cy.003:Cy.004:Cy.007:CyRPA, R5.015:R5.016:CyRPA), Soleil PROXIMA-1 (Cy.004:CyRPA, Cy.007:CyRPA) or Swiss Light Source PXIII (Cy.002:CyRPA). All data were collected under cryogenic conditions at 100 K. A data collection strategy was suggested by mosflm or EDNA^48,49^. Datasets were selected on the basis of superior resolution, overall completeness >95 %, and CC1/2 > 0.5. Data collection and refinement statistics are provided in Supplementary Table 4.

Phasing was done via molecular replacement using Phaser^50^, part of the CCP4 suite^51^. Two search models were used, one of CyRPA from PDB 5TIH and the other anti-RH5 mAb R5.016 (6RCS). In both cases, flexible regions such as CDR loops were removed from the search model prior to molecular replacement. IMGT was used to define the CDRs, which follows the system defined by Lefranc et al.^52^. Models were refined using Coot^53^, Phenix^54^, and Buster^55^ before validation with MolProbity^56^. While electron density for CyRPA, RH5, and antibody variable domains were clear and interpretable in all cases, in some of the structures, the electron density for some antibody constant domains was weak or missing, due to disorder. In such cases, a model of the constant domain was docked into the density, based on regions that were interpretable, and the occupancy of residues occupying regions of weak electron density were set to zero.

### Statistical analysis

Statistical analysis was done using Graphpad Prism v 9.1.0 for Mac. EC_50_ for GIA curves was determined through a four-parameter (max, min, variable Hill slope, EC_50_) logistic regression with the upper bound constrained to 100% GIA. A *p* value < 0.05 was considered significant.

### Reporting summary

Further information on research design is available in the Nature Research Reporting Summary linked to this article.

## Supplementary information

Supplementary Information

Reporting Summary

## Data Availability

Further information and requests for resources and reagents should be directed to and will be fulfilled by the lead contact, Simon J. Draper (simon.draper@bioch.ox.ac.uk). EURIPRED generated mAbs, Cy.003 (catalogued as 3B3#17), Cy.004 (4D12#30), Cy.007 (3A7#22) and Cy.009 (7B9#13) are available only through the National Institute of Biological Standards and Control (paul.bowyer@nibsc.org). Antibodies generated for this work will be shared on request, subject to a material transfer agreement. Crystallographic data is in the protein databank with accession codes 7PHU, 7PHV, 7PHW, 7PI2, 7PI3, and 7PI7. mAb sequence information is available in Supplementary Data Table 5. Source data are provided with this paper.

## Source data

Source Data
