# Supplementary material for: Heterotypic interactions drive antibody synergy against a malaria vaccine candidate
Source: Nat Commun. 2022 Feb 17;13:933. doi: 10.1038/s41467-022-28601-4 (PMC8854392; doi:10.1038/s41467-022-28601-4)
Supplement: Supplementary file 1 — Supplementary Information [file 41467_2022_28601_MOESM1_ESM.pdf]

Supplementary Information

Heterotypic interactions drive antibody synergy against a malaria vaccine candidate

Robert J. Ragotte, David Pulido, Amelia M. Lias, Doris Quinkert, Daniel G.W. Alanine, Abhishek Jamwal, Hannah Davies, Adéla Nacer, Edward D. Lowe, Geoffrey W. Grime, Joseph J. Illingworth, Robert F. Donat, Elspeth F. Garman, Paul W. Bowyer, Matthew K. Higgins*, Simon J. Draper*

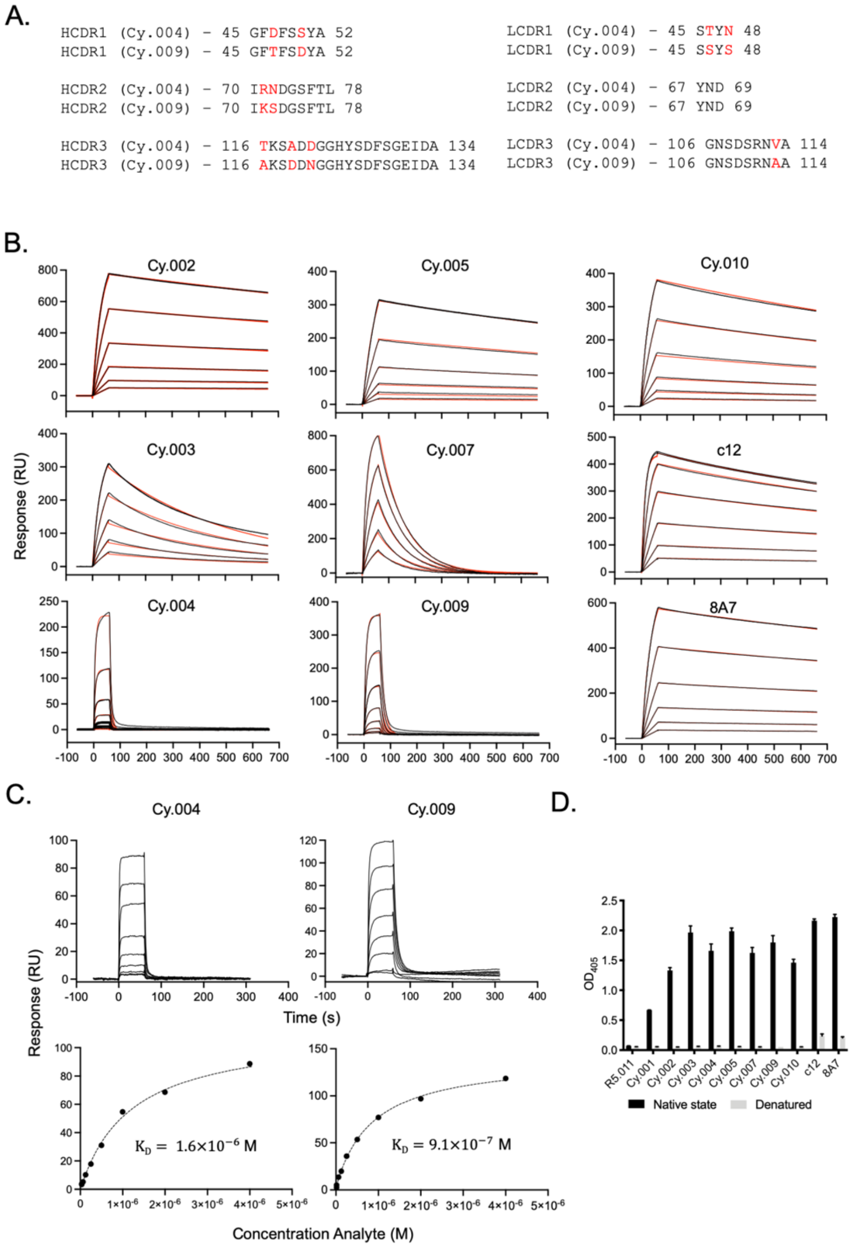

Supplementary Figure 1. (**A**) Comparison of CDR loops of Cy.004 and Cy.009 with amino acid changes indicated in red. (**B**) SPR sensorgrams of anti-CyRPA mAb binding to CyRPA across a 6-step 2-fold dilution series starting at either 125 nM for higher affinity mAbs (Cy.002, Cy.003, Cy.005, Cy.010, c12, 8A7) or 500 nM for lower affinity mAbs (Cy.004, Cy.007, Cy.009). (**C**) Steady state affinity determination of Cy.004 and Cy.009 from a 7-step 2-fold dilution series starting at 4 µM. (**D**) ELISA of mAb binding to native state or boiled and reduced CyRPA. Bars indicate the mean and error bars show the standard deviation across three replicates. R5.011 is an anti-RH5 mAb not expected to bind to CyRPA.

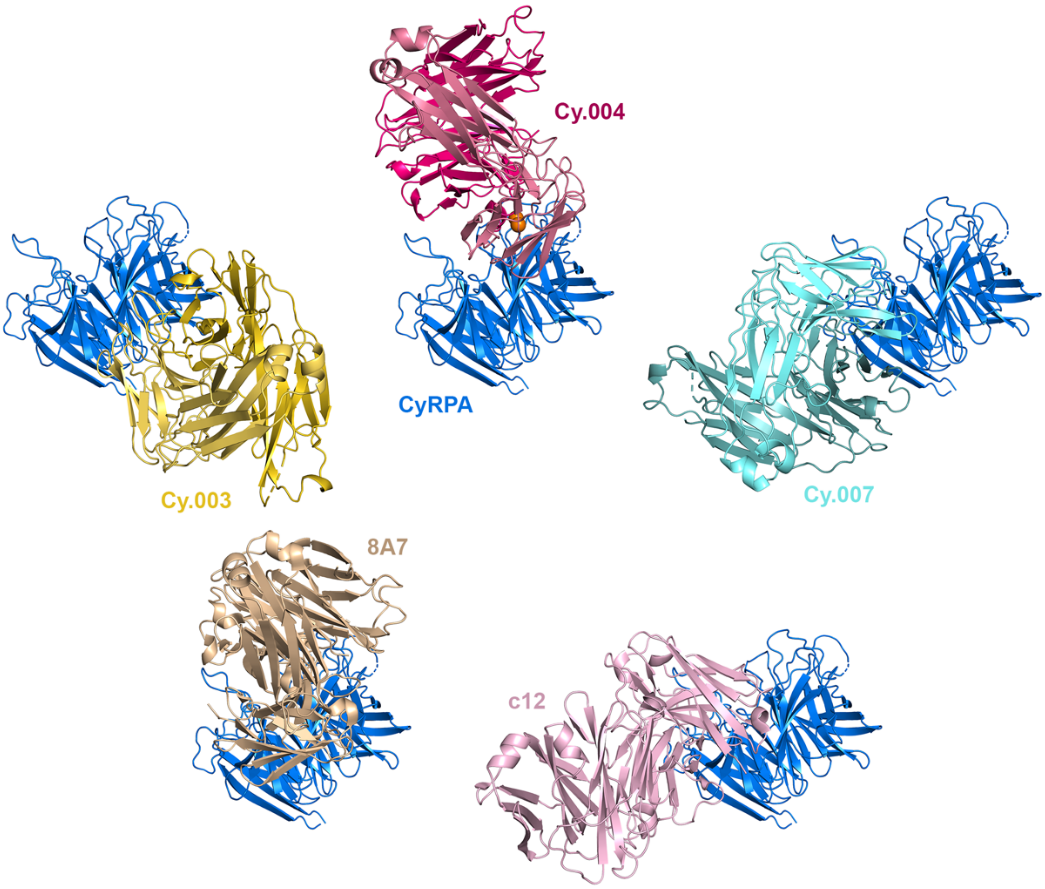

Supplementary Figure 2. Structures of CyRPA (blue) bound to growth inhibitory monoclonal antibodies. Cy.003 (yellow), Cy.004 (dark pink), and Cy.007 (light blue) are from this study while c12 (light pink) and 8A7 (gold) are from previous studies [22,26].

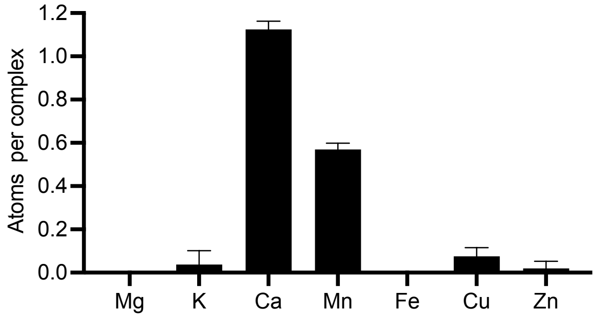

Supplementary Figure 3. MicroPIXE data showing the absolute abundance of different metal ions within the Cy.009:CyRPA complex. Bars indicate the mean and error bars show the standard deviation across three replicate measurements.

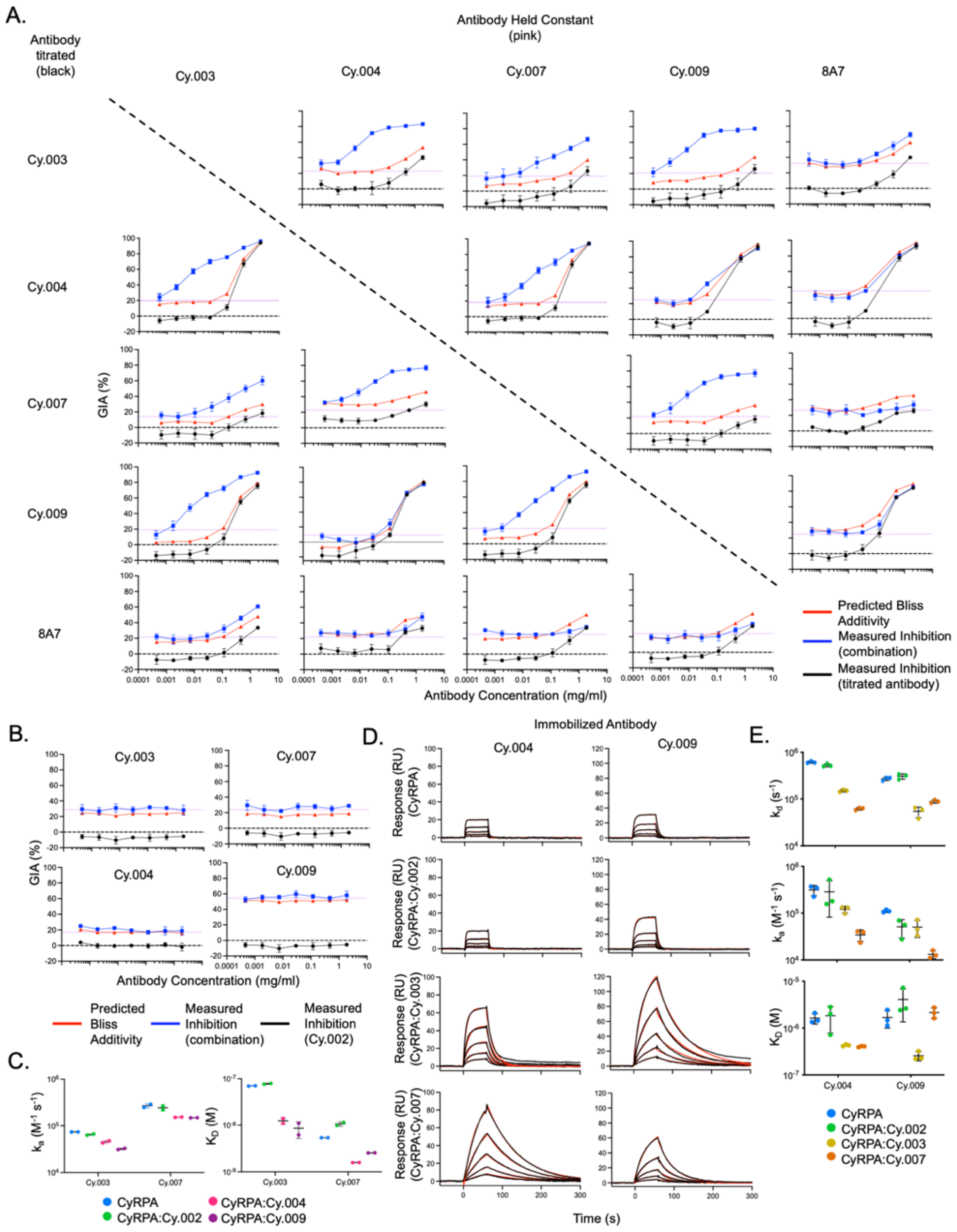

Supplementary Figure 4. *In vitro* GIA mAb synergy evaluations with one mAb titrated across a 7-step 4-fold dilution curve starting at 2 mg/ml with a second mAb held constant at ~20 % GIA (blue). Red indicates predicted Bliss additivity. Black indicates the inhibition curve of the titrated mAb alone from the same experiment. Pink dashed line indicates measured inhibition of the antibody held constant on its own. Each point is the mean across triplicates with error bars indicating the standard deviation. (**A**) Inhibitory mAb combinations. (**B**) mAbs in combination with Cy.002. (**C**) Summary of k_a and K_D changes of Cy.003 and Cy.007 binding to CyRPA and Fab:CyRPA complexes (representative sensorgrams in Figure 5C) across two replicate experiments with the mean and standard deviation shown by the horizontal line and error bars. (**D**) SPR sensorgrams of Cy.004 and Cy.009 binding to CyRPA compared to Fab:CyRPA complexes, all in the absence of $CaCl_2$. Black lines indicate the measured response while red lines indicate the curve fit. (**E**) Summary of k_d, k_a, and K_D changes of Cy.004 and Cy.009 binding to CyRPA and Fab:CyRPA complexes (representative sensorgrams in panel D) across three replicate experiments with the mean and standard deviation shown by the horizontal line and error bars.

Supplementary Table 1: Antibody binding kinetics summary table

mAb	Buffer	Ligand	Figure	k_a ($M^{-1}s^{-1}$)	k_d (s^{-1})	K_D (M)
Cy.002	PBS+P20	CyRPA	1	3.1×10^{5}	2.9×10^{-4}	9.4×10^{-10}
Cy.003	PBS+P20	CyRPA	1	1.2×10^{5}	4.7×10^{-3}	3.8×10^{-8}
Cy.004	PBS+P20	CyRPA	1	1.6×10^{5}	4.4×10^{-1}	2.8×10^{-6}
Cy.005	PBS+P20	CyRPA	1	1.4×10^{5}	4.1×10^{-4}	2.8×10^{-9}
Cy.007	PBS+P20	CyRPA	1	1.0×10^{5}	1.1×10^{-2}	1.1×10^{-7}
Cy.009	PBS+P20	CyRPA	1	1.0×10^{5}	1.5×10^{-1}	1.5×10^{-6}
Cy.010	PBS+P20	CyRPA	1	2.2×10^{5}	4.7×10^{-4}	2.1×10^{-9}
c12	PBS+P20	CyRPA	1	7.4×10^{5}	5.3×10^{-4}	7.2×10^{-10}
8A7	PBS+P20	CyRPA	1	2.5×10^{5}	2.9×10^{-4}	1.2×10^{-9}
Cy.004	TBS+P20	CyRPA	3	8.7×10^{4}	2.7×10^{-1}	3.1×10^{-6}
Cy.004	TBS+P20 (1mM $MgCl_2$)	CyRPA	3	6.9×10^{5}	1.7×10^{-1}	2.4×10^{-7}
Cy.004	TBS+P20 (1mM $CaCl_2$)	CyRPA	3	5.4×10^{5}	6.7×10^{-4}	1.2×10^{-9}
Cy.003	TBS+P20 (1mM $CaCl_2$)	CyRPA	5	7.4×10^{4}	5.1×10^{-3}	7.0×10^{-8}
Cy.003	TBS+P20 (1mM $CaCl_2$)	Cy.002:CyRPA	5	6.5×10^{4}	4.8×10^{-3}	7.7×10^{-8}
Cy.003	TBS+P20 (1mM $CaCl_2$)	Cy.004:CyRPA	5	4.8×10^{4}	5.7×10^{-4}	1.3×10^{-8}
Cy.003	TBS+P20 (1mM $CaCl_2$)	Cy.009:CyRPA	5	3.2×10^{4}	2.7×10^{-4}	8.6×10^{-9}
Cy.007	TBS+P20 (1mM $CaCl_2$)	CyRPA	5	2.9×10^{5}	1.6×10^{-3}	5.4×10^{-9}
Cy.007	TBS+P20 (1mM $CaCl_2$)	Cy.002:CyRPA	5	2.4×10^{5}	2.4×10^{-3}	1.1×10^{-8}
Cy.007	TBS+P20 (1mM $CaCl_2$)	Cy.004:CyRPA	5	1.5×10^{5}	2.4×10^{-4}	1.6×10^{-9}
Cy.007	TBS+P20 (1mM $CaCl_2$)	Cy.009:CyRPA	5	1.5×10^{5}	3.8×10^{-4}	2.6×10^{-9}
Cy.004	PBS+P20	CyRPA	S4	3.1×10^{5}	4.9×10^{-1}	1.3×10^{-6}
Cy.004	PBS+P20	Cy.002:CyRPA	S4	1.7×10^{5}	3.9×10^{-1}	1.8×10^{-6}
Cy.004	PBS+P20	Cy.003:CyRPA	S4	1.2×10^{5}	5.9×10^{-2}	4.1×10^{-7}
Cy.004	PBS+P20	Cy.007:CyRPA	S4	3.4×10^{4}	1.5×10^{-2}	3.9×10^{-7}
Cy.009	PBS+P20	CyRPA	S4	1.1×10^{5}	1.4×10^{-1}	8.9×10^{-7}
Cy.009	PBS+P20	Cy.002:CyRPA	S4	5.1×10^{4}	1.7×10^{-1}	1.5×10^{-6}
Cy.009	PBS+P20	Cy.003:CyRPA	S4	5.0×10^{4}	1.3×10^{-2}	2.6×10^{-7}
Cy.009	PBS+P20	Cy.007:CyRPA	S4	1.3×10^{4}	2.7×10^{-2}	2.1×10^{-6}

Supplementary Table 2: Interacting residues of mAb:CyRPA complexes.

CyRPA			Cy.002			
**Chain**	**Residue**	**Group**	**Chain**	**Residue**	**Group**	**Type of interaction**
A/D	Y185	Side chain	C/F	S51	Side chain	Hydrogen bond
A/D	K188	Side chain	B/E	D123	Side chain	Hydrogen bond
A/D	D189	Side chain	C/F	R74	Side chain	Hydrogen bond
A/D	K220	Side chain	B/E	T51	Main chain	Hydrogen bond
A/D	K220	Side chain	B/E	D71	Side chain	Hydrogen bond
A/D	L221	Main chain	B/E	D50	Main chain	Hydrogen bond
A/D	L221	Side chain	B/E	V119	Side chain	Hydrophobic
A/D	G222	Side chain	B/E	V119	Side chain	Hydrophobic
A/D	D245	Side chain	B/E	R69	Side chain	Hydrogen bond
A/D	D245	Main chain	C/F	R119	Side chain	Hydrogen bond
A/D	N246	Side chain	C/F	S115	Main chain	Hydrogen bond
A/D	N246	Main chain	C/F	D118	Main chain	Hydrogen bond
A/D	N248	Main chain	C/F	D118	Side chain	Hydrogen bond
**CyRPA**			**Cy.003**			
**Chain**	**Residue**	**Group**	**Chain**	**Residue**	**Group**	**Type of interaction**
A/D/G/J	R135	Side chain	B/E/H/K	D124	Main chain	Hydrogen bond
A/D/G/J	Y137	Side chain	C/F/I/L	Y49	Side chain	Hydrogen bond
A/D/G	E143	Side chain	C/F/I	K84	Side chain	Hydrogen bond
A/D/G/J	N145	Side chain	B/E/H/K	W125	Side chain	Hydrogen bond
A/D/G/J	N145	Side chain	C/F/I/L	S48	Side chain	Hydrogen bond
A/D/J	N146	Main chain	C/F/L	R109	Side chain	Hydrogen bond
A/D/G/J	D203	Side chain	B/E/H/K	S72	Side chain	Hydrogen bond
A/D/G/J	K204	Side chain	B/E/H/K	N121	Main chain	Hydrogen bond
A/D/G/J	E206	Side chain	B/E/H/K	S126	Side chain	Hydrogen bond
G/J	E206	Side chain	H/K	S126	Main chain	Hydrogen bond
**CyRPA**			**Cy.004**			
**Chain**	**Residue**	**Group**	**Chain**	**Residue**	**Group**	**Type of interaction**
A	E96	Main chain	C	Y47	Side chain	Hydrogen bond
A/D	D98	Side chain	B/E	S126	Main chain	Hydrogen bond
A/D	D98	Main chain	B/E	Y125	Main chain	Hydrogen bond
A/D	D98	Side chain	C/F	S108	Side chain	Hydrogen bond
A/D	L99	Main chain	C/F	R111	Side chain	Hydrogen bond
A/D	T100	Side chain	B/E	G122	Main chain	Hydrogen bond
A/D	T100	Main chain	B/E	G123	Main chain	Hydrogen bond
A/D	E120	Side chain	B/E	D120	Side chain	Metal coordination
A/D	E120	Side chain	B/E	D121	Side chain	Metal coordination
A/D	E122	Side chain	B/E	D121	Side chain	Metal coordination
A/D	N126	Side chain	B/E	S50	Main chain	Hydrogen bond
D	F94	Main chain	F	R111	Side chain	Hydrogen bond
D	T97	Main chain	F	R111	Side chain	Hydrogen bond
D	D98	Side chain	E	S126	Side chain	Hydrogen bond
**CyRPA**			**Cy.007**			
**Chain**	**Residue**	**Group**	**Chain**	**Residue**	**Group**	**Type of interaction**
A	K43	Side chain	B	D122	Side chain	Hydrogen bond
A	E96	Side chain	C	R108	Side chain	Hydrogen bond
A	K142	Side chain	B	T126	Side chain	Hydrogen bond
A/D	Y78	Side chain	B/E	R120	Side chain	Hydrogen bond
A/D	K81	Side chain	B/E	Y76	Side chain	Hydrogen bond
A/D	T89	Main chain	B/E	C124	Main chain	Hydrogen bond
A/D	D140	Main chain	B/E	Y76	Side chain	Hydrogen bond
D	N45	Side chain	E	S121	Main chain	Hydrogen bond
**RH5**			**R5.015**			
**Chain**	**Residue**	**Group**	**Chain**	**Residue**	**Group**	**Type of interaction**
A	K378	Side chain	B	D36	Side chain	Hydrogen bond
A	Q389	Side chain	B	F107	Main chain	Hydrogen bond
A	Q389	Side chain	B	F107	Main chain	Hydrogen bond
A	L393	Side chain	B	W106	Side chain	Hydrophobic
A	N396	Side chain	C	S31	Side chain	Hydrogen bond
A	N396	Side chain	C	D50	Side chain	Hydrogen bond
A	K400	Side chain	C	G28	Main chain	Hydrogen bond
A	K400	Side chain	C	D50	Side chain	Hydrogen bond
A	N491	Side chain	B	S110	Main chain	Hydrogen bond
A	F494	Side chain	B	W106	Side chain	Hydrophobic
A	F494	Side chain	B	P112	Side chain	Hydrophobic
A	Q502	Side chain	C	W90	Main chain	Hydrogen bond
A	Q502	Side chain	C	T92	Side chain	Hydrogen bond
A	N506	Side chain	C	N25	Side chain	Hydrogen bond

Supplementary Table 3: Heterotypic interactions between mAbs binding adjacent epitopes

**Cy.004**			**Cy.007**			
**Region**	**Residue**	**Group**	**Region**	**Residue**	**Group**	**Type of interaction**
LCDR3	D109	Side chain	LC Framework	S86	Side chain	Hydrogen bond
LCDR3	D109	Side chain	LC Framework	S86	Main chain	Hydrogen bond
LCDR3	S110	Side chain	LC Framework	K83	Side chain	Hydrogen bond
LCDR3	N112	Side chain	LC Framework	G85	Main chain	Hydrogen bond
**Cy.003**			**Cy.007**			
**Region**	**Residue**	**Group**	**Region**	**Residue**	**Group**	**Type of interaction**
LCDR1	G45	Main chain	LCDR3	N111	Side chain	Hydrogen bond
LC Framework	S87	Side chain	LCDR3	N112	Side chain	Hydrogen bond
LC Framework	T90	Side chain	HC framework	K84	Side chain	Hydrogen bond
**Cy.003**			**Cy.004**			
**Region**	**Residue**	**Group**	**Region**	**Residue**	**Group**	**Type of interaction**
HC framework	K84	Side chain	LC Framework	K83	Main chain	Hydrogen bond
LCDR3	N111	Side chain	LCDR1	T46	Side chain	Hydrogen bond
LCDR3	S112	Side chain	LC Framework	S84	Side chain	Hydrogen bond

Supplementary Table 4: Data collection and refinement statistics

	R5.015:R5.016:RH5	Cy.002:CyRPA	Cy.003:CyRPA	Cy.004:CyRPA	Cy.007:CyRPA	Cy.003:Cy.004: Cy.007:CyRPA
Space group	C 2 2 21	C 1 2 1	C 1 2 1	P 1 21 1	P 21 21 21	P 43
**Cell dimensions**						
a, b, c	97.74, 163.30, 176.82	205.05, 80.69, 117.36	354.30, 71.04, 164.79	103.06, 80.32, 114.91	74.10, 87.00, 342.77	164.84, 164.84, 382.30
α, β, γ (°)	90, 90, 90	90, 108.73, 90	90, 91.16, 90	90, 115.23, 90	90, 90, 90	90, 90, 90
Wavelength (Å)	0.9795	1.0000	0.97624	0.97857	0.97857	0.97950
Resolution (Å)	48.22 – 2.53 (2.57 – 2.53)	48.55 – 2.72 (2.81 – 2.72)	164.76 – 3.14 (3.21 – 3.14)	47.11 – 2.79 (2.87 – 2.79)	48.97 – 3.09 (3.22 – 3.09)	164.84 – 3.27 (3.28 – 3.27)
Total Observations	316074 (16521)	169805 (15709)	239064 (15017)	284862 (13827)	564609 (62331)	1115594 (11407)
Total Unique	47342 (2383)	49035 (4407)	72415 (4491)	42022 (2741)	41692 (4590)	156679 (1589)
R_{pim} (%)	4.1 (74.8)	4.7 (48.7)	13.6 (94.8)	10.4 (73.7)	14.4 (99.3)	4.9 (80.5)
R_{merge} (%)	9.9 (184.3)	4.9 (52.4)	13.9 (96.5)	16.7 (106.3)	35.7 (248.0)	12.0 (201.3)
R_{meas} (%)	10.7 (199.0)	6.8 (71.7)	19.5 (135.4)	19.7 (129.9)	38.5 (267.3)	12.9 (216.8)
$CC_{1/2}$	0.999 (0.575)	0.999 (0.864)	0.956 (0.425)	0.992 (0.757)	0.969 (0.733)	0.998 (0.586)
$I/\sigma(I)$	13.1 (1.2)	13.3 (1.8)	7.2 (1.3)	6.8 (0.9)	5.1(1.0)	9.4 (0.8)
Completeness (%)	99.6 (99.4)	99.6 (97.2)	99.9 (99.9)	99.9 (87.3)	99.9 (99.5)	99.9 (100.0)
Multiplicity	6.7 (6.9)	3.5 (3.6)	3.3 (3.3)	6.8 (5.0)	13.5 (13.6)	7.1 (7.2)
Wilson B factor (Å^2)	60	65	60	61	109	113.8
**Refinement**						
Reflections	47308	48960	72368	42008	41616	156578
R_{work} / R_{free}	23.6 / 27.6	27.0 / 29.8	21.9 / 23.9	23.9 / 27.7	28.7 / 33.2	28.5 / 29.8
Average B factor (Å^2)	63	99	89	90	176	187
**Number of residues**						
Protein	1172	1501	3002	1494	1476	6419
Water	127	76				
Ca^{2+}				4		8
**R.m.s deviations**						
Bond lengths (Å)	0.009	0.009	0.009	0.009	0.008	0.007
Bond angles (°)	1.04	1.07	1.06	1.06	0.95	0.90
**Ramachandran plot**						
Favored (%)	96.5	93.1	94.8	94.9	92.8	93.0
Allowed (%)	3.5	6.9	5.2	5.1	7.2	7.0
Outliers (%)	0	0	0	0	0.0	0

Values in parentheses indicate the highest resolution shell.

Supplementary Table 5: Monoclonal antibody sequences

mAb	Heavy Chain Sequence	Light Chain Sequence
Cy.002	MKCSWVIFFLMAVVSGVNSEVQLQQSGAELVK<br>PGASVKLSCTASGFNIKDTYIHWVKQRPEQGL<br>EWIGRIDPANGNTYSDPKFQDKATIKADTSSN<br>TAYLQLSSLTSEDTAVYYCARDVLYFDVWGAG<br>TTVTVSSASTKGPSVFPLAPSSKSTSGGTAAL<br>GCLVKDYFPEPVTVSWNSGALTSGVHTFPAVL<br>QSSGLYSLSSVVTVPSSSLGTQTYICNVNHKP<br>SNTKVDKKVEPKSCDKTHTCPPCPAPELLGGP<br>SVFLFPPKPKDTLMISRTPEVTCVVVDVSHED<br>PEVKFNWYVDGVEVHNAKTKPREEQYNSTYRV<br>VSVLTVLHQDWLNGKEYKCKVSNKALPAPIEK<br>TISKAKGQPREPQVYTLPPSRDELTKNQVSLT<br>CLVKGFYPSDIAVEWESNGQPENNYKTTPPVL<br>DSDGSFFLYSKLTVDKSRWQQGNVFSCSVMHE<br>ALHNHYTQKSLSLSPGK	METDTLLLWVLLLWVPGSTGDIVLTQ<br>SPASLAVSLGQRATISCRASESVDSY<br>GNSFMHWYQQKPGQPPKLLISRASNL<br>ESGIPARFSGSGSRTDFTLTINPVEA<br>DDVATYYCQQSNEDRTFGGGTKLEIE<br>RTVAAPSVFIFPPSDEQLKSGTASVV<br>CLLNNFYPREAKVQWKVDNALQSGNS<br>QESVTEQDSKDSTYSLSSTLTLSKAD<br>YEKHKVYACEVTHQGLSSPVTKSFNR<br>GEC
Cy.003	MEWSWVFLFFLSVTTGVHSAVTLDESGGGLQT<br>PGGALSLVCKGSGFFSFSSYTMQWVRQAPGKG<br>LEWVASISSGGGTNYGAAVKGRATISRDNGQS<br>TLRLQLNNLRAEDTGTYYCAKHGVNGCDWSYS<br>VGCVDAWGHGTEVIVSSASTKGPSVFPLAPSS<br>KSTSGGTAALGCLVKDYFPEPVTVSWNSGALT<br>SGVHTFPAVLQSSGLYSLSSVVTVPSSSLGTQ<br>TYICNVNHKPSNTKVDKKVEPKSCDKTHTCPP<br>CPAPELLGGPSVFLFPPKPKDTLMISRTPEVT<br>CVVVDVSHEDPEVKFNWYVDGVEVHNAKTKPR<br>EEQYNSTYRVVSVLTVLHQDWLNGKEYKCKVS<br>NKALPAPIEKTISKAKGQPREPQVYTLPPSRD<br>ELTKNQVSLTCLVKGFYPSDIAVEWESNGQPE<br>NNYKTTPPVLDSDGSFFLYSKLTVDKSRWQQG<br>NVFSCSVMHEALHNHYTQKSLSLSPGK	MSVPTQVLGLLLLWLTDARCALTQPS<br>SVSANPGETVKITCSGGSSSYYGWYQ<br>QKSPGSAPVTLIYNNQKRPSDIPSRF<br>SGSKSGSTGTLTITGVQAEDEAVYFC<br>GSRDNSGGIFGAGTTLTVLRTVAAPS<br>VFIFPPSDEQLKSGTASVVCLLNNFY<br>PREAKVQWKVDNALQSGNSQESVTEQ<br>DSKDSTYSLSSTLTLSKADYEKHKVY<br>ACEVTHQGLSSPVTKSFNRGEC
Cy.004	MEWSWVFLFFLSVTTGVHSAVTLDESGGGLQT<br>PGGALSLVCKASGFDFSSYAMGWVRQAPGKGL<br>EYVAGIRNDGSFTLYTPAVKGRATISRDNGQS<br>TVRLQLNNLRAEDTATYFCTKSADDGGHYSDF<br>SGEIDAWGHGTEVIVSSASTKGPSVFPLAPSS<br>KSTSGGTAALGCLVKDYFPEPVTVSWNSGALT<br>SGVHTFPAVLQSSGLYSLSSVVTVPSSSLGTQ<br>TYICNVNHKPSNTKVDKKVEPKSCDKTHTCPP<br>CPAPELLGGPSVFLFPPKPKDTLMISRTPEVT<br>CVVVDVSHEDPEVKFNWYVDGVEVHNAKTKPR<br>EEQYNSTYRVVSVLTVLHQDWLNGKEYKCKVS<br>NKALPAPIEKTISKAKGQPREPQVYTLPPSRD<br>ELTKNQVSLTCLVKGFYPSDIAVEWESNGQPE<br>NNYKTTPPVLDSDGSFFLYSKLTVDKSRWQQG<br>NVFSCSVMHEALHNHYTQKSLSLSPGK	MSVPTQVLGLLLLWLTDARCALTQPS<br>SVSANPGETVKITCSGSTYNYGWYQQ<br>KSPGSAPVTVIYYNDKRPSDIPSRFS<br>GSKSGSTGTLTITGVQAEDEAVYYCG<br>NSDSRNVAFGAGTTLTVLRTVAAPSV<br>FIFPPSDEQLKSGTASVVCLLNNFYP<br>REAKVQWKVDNALQSGNSQESVTEQD<br>SKDSTYSLSSTLTLSKADYEKHKVYA<br>CEVTHQGLSSPVTKSFNRGEC
Cy.005	MNFGLSLIFLALILKGVQCEVQLVESGGDLVK<br>PGGSLKLSCAASGFTFSNYGLSWVRQTPDKRL<br>EWVAAISSGGSYTYYPDSVKGRFTISRDNAKN<br>TLYLQMSSLKSADTAMYYCARHTGSHRYAWFT<br>YWGQGTLVTVSAASTKGPSVFPLAPSSKSTSG<br>GTAALGCLVKDYFPEPVTVSWNSGALTSGVHT<br>FPAVLQSSGLYSLSSVVTVPSSSLGTQTYICN<br>VNHKPSNTKVDKKVEPKSCDKTHTCPPCPAPE<br>LLGGPSVFLFPPKPKDTLMISRTPEVTCVVVD<br>VSHEDPEVKFNWYVDGVEVHNAKTKPREEQYN<br>STYRVVSVLTVLHQDWLNGKEYKCKVSNKALP	MAWTPLFFFFVLHCSGSFSQLVLTQS<br>SSASFSLGASAKLTCTLSSQHSTYTI<br>EWYQQQPLKPPKYVMELKKDGSHSTG<br>DGIPDRFSGSSSGADRYLSISNIQPE<br>DEAIYICGVVDTIKEQFVYVFGGGTK<br>VTVLRTVAAPSVFIFPPSDEQLKSGT<br>ASVVCLLNNFYPREAKVQWKVDNALQ<br>SGNSQESVTEQDSKDSTYSLSSTLTL<br>SKADYEKHKVYACEVTHQGLSSPVTK<br>SFNRGEC

	APIEKTISKAKGQPREPQVYTLPPSRDELTKN<br>QVSLTCLVKGFYPSDIAVEWESNGQPENNYKT<br>TPPVLDSDGSFFLYSKLTVDKSRWQQGNVFSC<br>SVMHEALHNHYTQKSLSLSPGK	
Cy.007	MEWSWVFLFFLSVTTGVHSAVTLDESGGGLQT<br>PGGTLSLVCKGSGFTFSSYEMQWVRQAPGKGL<br>EWVADICIGGSYTGYGPAVKGRATISRDDGQS<br>TVRLQLNNLRAEDTGTYYCAKTARSDYCITGL<br>DDIDAWGHGTEVIVSSASTKGPSVFPLAPSSK<br>STSGGTAALGCLVKDYFPEPVTVSWNSGALTS<br>GVHTFPAVLQSSGLYSLSSVVTVPSSSLGTQT<br>YICNVNHKPSNTKVDKKVEPKSCDKTHTCPPC<br>PAPELLGGPSVFLFPPKPKDTLMISRTPEVTC<br>VVVDVSHEDPEVKFNWYVDGVEVHNAKTKPRE<br>EQYNSTYRVVSVLTVLHQDWLNGKEYKCKVSN<br>KALPAPIEKTISKAKGQPREPQVYTLPPSRDE<br>LTKNQVSLTCLVKGFYPSDIAVEWESNGQPEN<br>NYKTTPPVLDSDGSFFLYSKLTVDKSRWQQGN<br>VFSCSVMHEALHNHYTQKSLSLSPGK	MSVPTQVLGLLLLWLTDARCALTQPS<br>SVSANPGETVKITCSGGSSDYGWYQQ<br>KSPGSAPVTVIYENNKRPSDIPSRFS<br>GSKFGSTHTLTITGVQADDEAVYFCG<br>SRDTNNGAFGAGTTLTVLRTVAAPSV<br>FIFPPSDEQLKSGTASVVCLLNNFYP<br>REAKVQWKVDNALQSGNSQESVTEQD<br>SKDSTYSLSSTLTLSKADYEKHKVYA<br>CEVTHQGLSSPVTKSFNRGEC
Cy.009	MEWSWVFLFFLSVTTGVHSAVTLDESGGGLQT<br>PGGALSLVCKASGFTFSDYAMGWVRQAPGKGL<br>EYVAGIKSDGSFTLYGSTVKGRATISRDNGQS<br>IVRLQLNNLRAEDTGTYYCAKSDDNGGHYSDF<br>SGEIDAWGHGTEVIVSSASTKGPSVFPLAPSS<br>KSTSGGTAALGCLVKDYFPEPVTVSWNSGALT<br>SGVHTFPAVLQSSGLYSLSSVVTVPSSSLGTQ<br>TYICNVNHKPSNTKVDKKVEPKSCDKTHTCPP<br>CPAPELLGGPSVFLFPPKPKDTLMISRTPEVT<br>CVVVDVSHEDPEVKFNWYVDGVEVHNAKTKPR<br>EEQYNSTYRVVSVLTVLHQDWLNGKEYKCKVS<br>NKALPAPIEKTISKAKGQPREPQVYTLPPSRD<br>ELTKNQVSLTCLVKGFYPSDIAVEWESNGQPE<br>NNYKTTPPVLDSDGSFFLYSKLTVDKSRWQQG<br>NVFSCSVMHEALHNHYTQKSLSLSPGK	MSVPTQVLGLLLLWLTDARCALTQPS<br>SVSANPGETVKITCSGSSYSYGWYQQ<br>KSPGSAPVTVIYYNDKRPSDIPSRFS<br>GSKSGSTGTLTITGVQAEDEAVYFCG<br>NSDSRNAAFGAGTTLTVLRTVAAPSV<br>FIFPPSDEQLKSGTASVVCLLNNFYP<br>REAKVQWKVDNALQSGNSQESVTEQD<br>SKDSTYSLSSTLTLSKADYEKHKVYA<br>CEVTHQGLSSPVTKSFNRGEC
Cy.010	MNLGLSFIFLALILKGVQCEVQLVESGGGLVQ<br>PGGSLKLSCAASGFTFSTYGMSWVRQTPDKRL<br>ELVATINSNGGNTYYPDSVKGRFTMSRDNAKN<br>TLYLQMSSLKSEDTAMYYCAREGYYYGGYSYA<br>MDYWGQGTSVTVSSASTKGPSVFPLAPSSKST<br>SGGTAALGCLVKDYFPEPVTVSWNSGALTSGV<br>HTFPAVLQSSGLYSLSSVVTVPSSSLGTQTYI<br>CNVNHKPSNTKVDKKVEPKSCDKTHTCPPCPA<br>PELLGGPSVFLFPPKPKDTLMISRTPEVTCVV<br>VDVSHEDPEVKFNWYVDGVEVHNAKTKPREEQ<br>YNSTYRVVSVLTVLHQDWLNGKEYKCKVSNKA<br>LPAPIEKTISKAKGQPREPQVYTLPPSRDELT<br>KNQVSLTCLVKGFYPSDIAVEWESNGQPENNY<br>KTTPPVLDSDGSFFLYSKLTVDKSRWQQGNVF<br>SCSVMHEALHNHYTQKSLSLSPGK	MLHRDMNMLTQLLGLLLLWFAGGKCD<br>IQMTQSPASQSASLGESVTITCLASQ<br>TIGIWLAWYQQKPGKSPQLLIYAASS<br>LADGVPSRFSGSGSGTKFSFKISSLQ<br>AEDFVSYYCQQLYSTPWTFGGGTKLE<br>IKRTVAAPSVFIFPPSDEQLKSGTAS<br>VVCLLNNFYPREAKVQWKVDNALQSG<br>NSQESVTEQDSKDSTYSLSSTLTLSK<br>ADYEKHKVYACEVTHQGLSSPVTKSF<br>NRGEC
