# Supplementary material for: Heterotypic interactions drive antibody synergy against a malaria vaccine candidate
Source: Nat Commun. 2022 Feb 17;13:933. doi: 10.1038/s41467-022-28601-4 (PMC8854392; doi:10.1038/s41467-022-28601-4)
Supplement: Supplementary file 2 — Reporting Summary [file 41467_2022_28601_MOESM2_ESM.pdf]

nature portfolio

Corresponding author(s): Matthew K. Higgins and Simon J. Draper

Last updated by author(s): Jan 18, 2022

Reporting Summary

Nature Portfolio wishes to improve the reproducibility of the work that we publish. This form provides structure for consistency and transparency in reporting. For further information on Nature Portfolio policies, see our Editorial Policies and the Editorial Policy Checklist.

Statistics

For all statistical analyses, confirm that the following items are present in the figure legend, table legend, main text, or Methods section.

n/a	Confirmed	
☐	☒	The exact sample size (*n*) for each experimental group/condition, given as a discrete number and unit of measurement
☐	☒	A statement on whether measurements were taken from distinct samples or whether the same sample was measured repeatedly
☐	☒	The statistical test(s) used AND whether they are one- or two-sided<br>*Only common tests should be described solely by name; describe more complex techniques in the Methods section.*
☒	☐	A description of all covariates tested
☒	☐	A description of any assumptions or corrections, such as tests of normality and adjustment for multiple comparisons
☐	☒	A full description of the statistical parameters including central tendency (e.g. means) or other basic estimates (e.g. regression coefficient) AND variation (e.g. standard deviation) or associated estimates of uncertainty (e.g. confidence intervals)
☐	☒	For null hypothesis testing, the test statistic (e.g. *F*, *t*, *r*) with confidence intervals, effect sizes, degrees of freedom and *P* value noted<br>*Give P values as exact values whenever suitable.*
☒	☐	For Bayesian analysis, information on the choice of priors and Markov chain Monte Carlo settings
☒	☐	For hierarchical and complex designs, identification of the appropriate level for tests and full reporting of outcomes
☒	☐	Estimates of effect sizes (e.g. Cohen's *d*, Pearson's *r*), indicating how they were calculated

Our web collection on statistics for biologists contains articles on many of the points above.

Software and code

Policy information about availability of computer code

Data collection	For collection of SPR data, we used the 'Biacore X100 control' software.
Data analysis	For analysis of SPR data, we used the 'Biacore X100 evalutation tools'. For analysis of crystallographic data, we used XDS, Xia2, Phaser 2.8.3, Buster, PHENIX 1.13-2998 and COOT v0.8.9.2. Visualization was done with PyMol v2.3.3.All are standard and are freely available for academic use. For analysis of SPR data, we used the Biacore evaluation software, as provided with the instrument. No bespoke software was used.

For manuscripts utilizing custom algorithms or software that are central to the research but not yet described in published literature, software must be made available to editors and reviewers. We strongly encourage code deposition in a community repository (e.g. GitHub). See the Nature Portfolio guidelines for submitting code & software for further information.

Data

Policy information about availability of data

All manuscripts must include a data availability statement. This statement should provide the following information, where applicable:

- Accession codes, unique identifiers, or web links for publicly available datasets
- A description of any restrictions on data availability
- For clinical datasets or third party data, please ensure that the statement adheres to our policy

Further information and requests for resources and reagents should be directed to and will be fulfilled by the lead contact, Simon J. Draper (simon.draper@bioch.ox.ac.uk). EURIPRED generated mAbs, Cy.003 (catalogued as 3B3#17), Cy.004 (4D12#30), Cy.007 (3A7#22) and Cy.009 (7B9#13) are available only through the National Institute of Biological Standards and Control (paul.bowyer@nibsc.org). Antibodies generated for this work will be shared on request, subject to a material transfer agreement. Crystallographic data and models are in the protein databank with accession codes 7PHU, 7PHV, 7PHW, 7PI2, 7PI3 and 7PI6. mAb sequence information is available in Supplementary Data Table 5.

Field-specific reporting

Please select the one below that is the best fit for your research. If you are not sure, read the appropriate sections before making your selection.

- [x] Life sciences
- [ ] Behavioural & social sciences
- [ ] Ecological, evolutionary & environmental sciences

For a reference copy of the document with all sections, see nature.com/documents/nr-reporting-summary-flat.pdf

Life sciences study design

All studies must disclose on these points even when the disclosure is negative.

Sample size	The sample size for determining the activity of the antibodies described here was based on the principles of running all assays in triplicate and repeating experiments to obtain multiple experimental replicates. For mouse immunizations to generate Cy.002, Cy.005, and Cy.010, two mice were immunized as this was sufficient to generate the biological material (spleens of immunized mice) that would be used for the antibody isolation. Since no conclusions were being drawn from the mouse response to immunization, two was sufficient for the purposes of isolating antibodies.
Data exclusions	No data was excluded
Replication	All experiments were repeated at least twice to ensure reproducibility of the data. Replication criteria for any given assay is described in its associated figure legend.
Randomization	Randomization was not relevant to this study as no subjective judgements were required about which data to include, exclude or measure.
Blinding	Researchers involved in the acquisition of growth-inhibitory activity (GIA) data in which the researchers conducting the experiments did not know the outcome of other studies on the same antibodies. All other experiments were performed with the knowledge of the antibody name.

Reporting for specific materials, systems and methods

We require information from authors about some types of materials, experimental systems and methods used in many studies. Here, indicate whether each material, system or method listed is relevant to your study. If you are not sure if a list item applies to your research, read the appropriate section before selecting a response.

Materials & experimental systems

n/a	Involved in the study	
☐	☒	Antibodies
☐	☒	Eukaryotic cell lines
☒	☐	Palaeontology and archaeology
☐	☒	Animals and other organisms
☒	☐	Human research participants
☒	☐	Clinical data
☒	☐	Dual use research of concern

Methods

n/a	Involved in the study	
☒	☐	ChIP-seq
☒	☐	Flow cytometry
☒	☐	MRI-based neuroimaging

Antibodies

Antibodies used	Antibodies Cy.002-010 were generated in this study. Antibodies c12, 8A7, and R5.015 are described in other studies and in each case the citation is provided in the manuscript. The anti-human IgG alkaline phosphatase secondary antibody used was purchased from Sigma-Aldrich (A3187)
Validation	Validation of Cy.002-010 by ELISA, structural biology, surface plasmon resonance and growth inhibitory assays is a major part of this work and the data is presented. c12 and 8A7 were validated for binding to CyRPA through ELISA and SPR. R5.015 was previously validated for binding through ELISA and SPR (Alanine et al. 2019) and here it was further validated by crystallography.

Eukaryotic cell lines

Policy information about cell lines

Cell line source(s)	HEK293F cells used for protein expression were obtained from ThermoFisher. However, these were not studied to dereive experimental data. Instead they were used to purify protein which was validated and was studied to obtain data.
Authentication	HEK293F cells were not authenticated.
Mycoplasma contamination	HEK293F cells were not tested for mycoplasma contamination.

Commonly misidentified lines (See ICLAC register)	N/A

Animals and other organisms

Policy information about studies involving animals; ARRIVE guidelines recommended for reporting animal research

Laboratory animals	Two female BALC/c mice were immunised with CyRPA to allow the generation of a panel of monoclonal antibodies and their spleens were harvested for this purpose. Animals were used as this allows the generation of high quality affinity matured antibodies. The animals were housed in individual ventilated cages, with a light and dark cycle of 12h each. The temperature was maintained at 20-24ºC.
Wild animals	This study did not involve wild animals.
Field-collected samples	This study did not involve samples collected from the field.
Ethics oversight	As described in the manuscript: "All procedures on mice were performed in accordance with the terms of the UK Animals (Scientific Procedures) Act Project Licence (PA7D20B85) and were approved by the University of Oxford Animal Welfare and Ethical Review Body."

Note that full information on the approval of the study protocol must also be provided in the manuscript.
